# Chemical Characterization of Secondary Metabolites of *Escovopsioides nivea* and Their Antagonistic Activity Against the Leaf-Cutter Ant Fungal Mutualist *Leucoagaricus gongylophorus*

**DOI:** 10.3390/molecules31213847

**Published:** 2026-10-25

**Authors:** Tania Pérez, Belén Z. Hernández, Eyleen Vega Gibbs, Ricardo Santamaría, Daniel Torres-Mendoza, Marc G. Chevrette, Hermógenes Fernández-Marín, Humberto E. Ortega

**Affiliations:** 1 Laboratorio de Bioorgánica Tropical, Facultad de Ciencias Naturales, Exactas y Tecnología, Universidad de Panamá, Panama 0824, Panama; tania.perez-t@up.ac.pa (T.P.); belen.hernandez@up.ac.pa (B.Z.H.); daniel-t.torres-m@up.ac.pa (D.T.-M.); 2 Escuela de Biología, Facultad de Ciencias Naturales, Exactas y Tecnología, Universidad de Panamá, Panama 0824, Panama; eyleen.vega-g@up.ac.pa; 3 División de Bioprospección y Descubrimiento de Fármacos, Instituto de Investigaciones Científicas y Servicios de Alta Tecnología (INDICASAT AIP), Panama 0843-01103, Panama; rsantamaria@indicasat.org.pa; 4 Vicerrectoría de Investigación y Postgrado, Universidad de Panamá, Panama 0824, Panama; 5 Department of Plant Pathology and Wisconsin Institute for Discovery, University of Wisconsin-Madison, Madison, WI 53715, USA; chevrette@wisc.edu; 6 División de Biodiversidad, Instituto de Investigaciones Científicas y Servicios de Alta Tecnología (INDICASAT AIP), Panama 0843-01103, Panama; 7 Sistema Nacional de Investigación (SNI), Secretaría Nacional de Ciencia, Tecnología e Innovación, Ciudad del Saber, Clayton, Panama 0816, Panama; 8 Departamento de Química Orgánica, Facultad de Ciencias Naturales, Exactas y Tecnología, Universidad de Panamá, Panama 0824, Panama

**Keywords:** *Acromyrmex*, *Escovopsioides*, *Leucoagaricus gongylophorus*, symbiosis, fungus-growing ants, secondary metabolites

## Abstract

Microfungi of the genus *Escovopsioides* are antagonists within the obligate symbiosis between fungus-growing ants (Tribe Attini) and their fungal cultivars, yet the chemical basis of their interactions remains largely unexplored. Similar to species of the closely related genus *Escovopsis*, *Escovopsioides* species frequently exhibit antagonistic interactions with the basidiomycete *Leucoagaricus gongylophorus*, the fungal cultivar maintained by attine ants. In this study, *Escovopsioides nivea* strains were isolated from nests of the higher attine ants *Acromyrmex octospinosus* and *Mycetomoellerius mikromelanos* collected in central Panama. One strain isolated from an *A. octospinosus* nest was selected for chemical investigation and evaluation of its antagonistic activity against *L. gongylophorus*. Chemical analysis using one- and two-dimensional NMR spectroscopy in combination with electrospray ionization mass spectrometry (ESIMS) led to the identification of five known secondary metabolites from *Escovopsioides nivea*: rugulotrosin A (**1**), microthecin (**2**), 2-(1-oxo-2-hydroxyethyl)furan (**3**), chrysophanol (**4**), and physcion (**5**). Bioassays against *L. gongylophorus* revealed that rugulotrosin A (**1**), chrysophanol (**4**), and physcion (**5**) exhibited moderate antagonistic activity at 100 μg concentrations. To the best of our knowledge, this is the first report describing secondary metabolites produced by a member of the genus *Escovopsioides* and demonstrating their antagonistic activity against the mutualistic fungal cultivar of fungus-growing ants. These findings broaden the known chemical diversity of *Escovopsioides* and provide new insights into the ecological functions and evolutionary significance of microfungal antagonists associated with the fungus-growing ant symbiosis.

## 1. Introduction

Secondary metabolites are specialized bio-active compounds synthesized by bacteria, fungi, and plants in response to specific environmental stimuli [1,2]. Unlike primary metabolites, these natural products play essential roles in mediating the complex ecological dynamics of symbiotic systems, drive interspecies interactions, and shape evolutionary trajectories [3,4,5]. Consequently, deciphering the chemical interactions that govern microbial community dynamics within complex symbiotic networks is crucial for illuminating the ecological and evolutionary mechanisms underpinning such mutualistic and parasitic associations [4].

Originating approximately 66 million years ago, the obligate mutualism between fungus-growing attine ants (Tribe Attini) and their cultivated basidiomycete fungi represents a deeply integrated evolutionary partnership [6,7,8,9]. Within this mutualism, the ants provide physical protection, nutrients, and dispersal for the cultivar, while the fungus serves as the primary, if not sole, nutritional source for both the brood and adult ants [6,7,8,9]. Over evolutionary time, this classic bipartite system has evolved into a highly complex, multi-trophic symbiotic network involving at least three additional major microbial cohorts, including: (i) an specialized actinobacteria (e.g., *Pseudonocardia* spp.) housed on the ant cuticle, which produce targeted secondary metabolites that selectively inhibit specialized microparasites of the cultivar [10,11]; (ii) virulent microparasitic fungi belonging to the genus *Escovopsis*, which threaten the cultivar [10,12,13,14] as well as other genera member of the *Escovopsis* group (*Luteomycetes*, *Sympodiorose, Manidigitorum* and *Escovopsioides*) [15,16,17,18,19]: and (iii) microparasitic black yeasts that colonize the ant cuticle, antagonize the protective actinobacteria, and thereby can negatively impact host chemical defenses [20].

The integrity and stability of this multi-species symbiosis are fundamentally dictated by a continuous chemical warfare operating among all symbionts: actinobacteria, microparasites (*Escovopsis* spp.), cultivated basidiomycetes, and attine ants (including the use of glandular antimicrobial secretions of phenylacetic acid produced directly by the ants which provide additional defensive barriers against parasite infestation) [13,21,22]. In this ongoing evolutionary arms race, *Escovopsis* strains secrete specialized secondary metabolites that can directly inhibit actinobacterial growth, while simultaneously producing bioactive compounds that can impair ant behavior and survival [23,24].

Although the interactions between microparasitic fungi and the fungal cultivar were historically viewed direct parasitism, growing evidence indicates that many associated lineages exhibit broader antagonistic relationships [18,19,21,25,26,27,28,29,30]. Fungal genera frequently isolated from attine nests that display antagonistic activity against the cultivated basidiomycete include *Escovopsis*, *Sympodiorosea*, *Luteomyces*, *Manidigitorum*, and *Escovopsioides* [19,20,21]. Despite the recognized prevalence of these antagonistic interactions, the specific bioactive secondary metabolites responsible for driving these antagonistic responses remain largely uncharacterized [31].

Among these nest-associated fungi, the genus *Escovopsioides* is frequently isolated from the colonies of diverse fungus-farming ant species, particularly across South America [22]. While *Escovopsioides* species are known to strongly antagonize the basidiomycete cultivar (i.e., *Leucoagaricus gongylophorus*), their exact trophic role, whether strictly antagonistic or parasitic, remains a subject of investigation. *Escovopsioides* shares key morphological traits with *Escovopsis*, but it is distinctively differentiated by its terminal vesicles bearing elongated, lageniform phialides and hyphae producing specialized aleuroconidia [18,19,32,33]. To date, the genus comprises a single formally described species, *Escovopsioides nivea*, whose detailed ecological function within leaf-cutting ant colonies remains incompletely understood [23]. Although bioassays have confirmed that *E. nivea* inhibits the growth of *L. gongylophorus* [23,24], the underlying chemical mediators have not been identified.

In this study, we report for the first time the isolation, structural characterization, and identification of secondary metabolites produced by *E. nivea*. Furthermore, we evaluate the bioactivity of these isolated natural products in bioassays against *L. gongylophorus* to elucidate their potential role in driving antagonistic interactions within attine ant symbiotic communities.

## 2. Results and Discussion

### 2.1. Prevalence of Escovopsioides in Fungus Gardens of Attine Ant Nests

**Figure 1:**
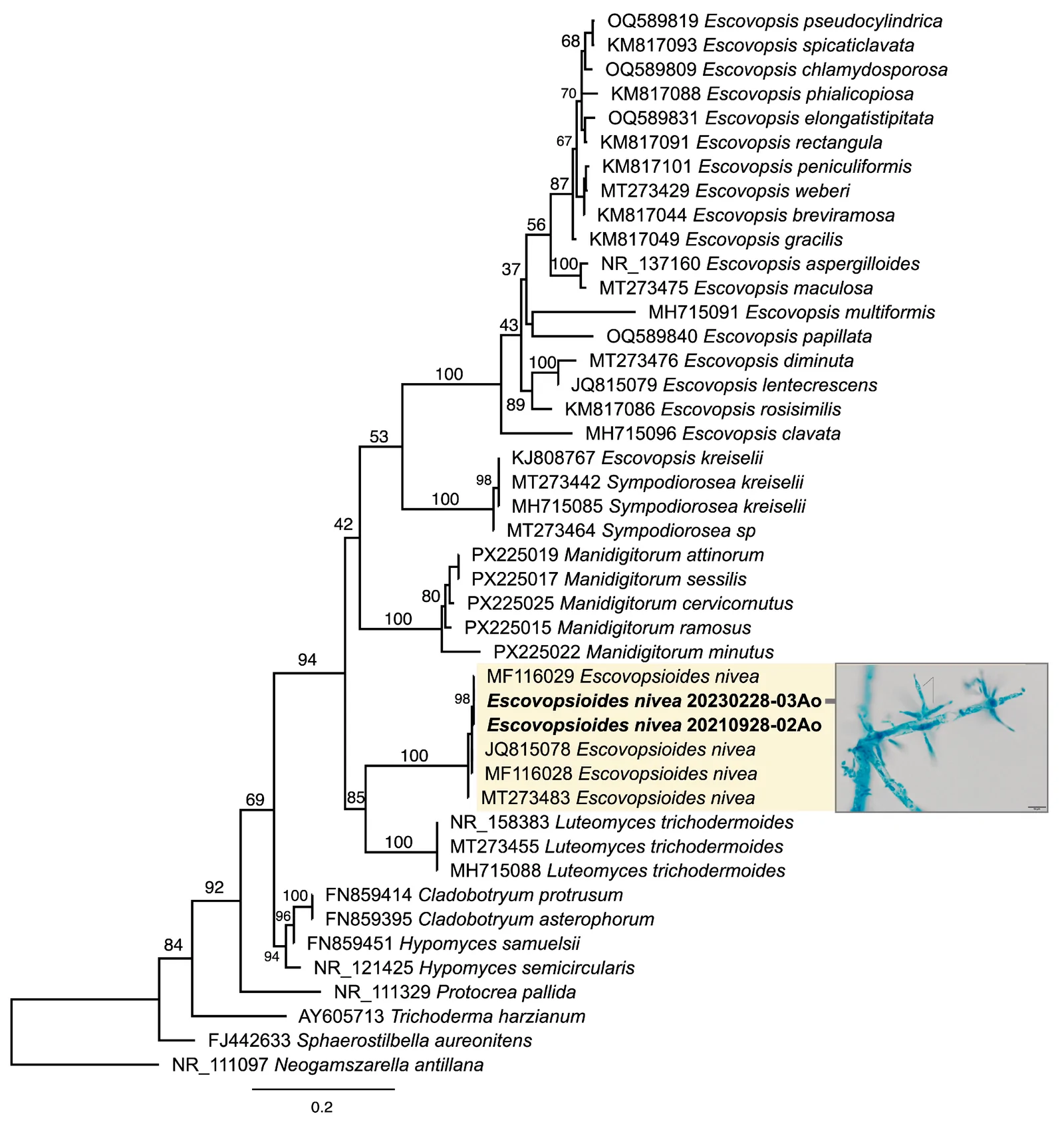
*Escovopsis* group phylogeny with reference to *E. nivea* isolated from Panama, marked on the *E. nivea* clade.

*E. nivea* was observed in 10% in *Acromyrmex octospinosus* nests (2 of 20 nests) and 12.5% in *Mycetomoellerius mikromelanos* nests (1 of 8 nests). These prevalence rates are lower compared with Augustin et al. [32], who isolated *E. nivea* in 14 of 25 colonies (56%) collected from three *Acromyrmex* species in Brazil. Mendonça et al. [33] observed a similarly low prevalence of 8.3% in 12 colonies collected in Brazil. In addition, Pietrobon et al. [26] report an extensive occurrence of *E. nivea* fungus in nests collected across six distinct habitats within eight locations in Brazil, encompassing both higher attines (leaf-cutting ants and non-leaf-cutting attines). Furthermore, Reis et al. [34] noted that *E. nivea* was the second most frequently isolated fungus from 12 sampled colonies in Brazil. Overall, our findings expand the known geographic distribution of *Escovopsioides* [19,26,33,34], and suggest that *E. nivea* is consistently observed on the fungus garden of attine nests. The morphological characteristics of *Escovopsioides*, particularly its hyaline mycelium and absence of pigmentation, represent the key features distinguishing it from *Escovopsis* [33,34]. Species identification in this study was established using the diagnostic morphological traits described herein, alongside molecular characterization, which confirmed the sequence identity for the isolate obtained from *A. octospinosus* (Figure 1). The slow growth rate of this microfungus poses a significant challenge for its isolation and culturing. In a study by Ostis and Rodrigues [19], exogenous microorganisms associated with ant nests were shown to inhibit the growth of *Escovopsioides* in culture media, providing a plausible explanation for the variable prevalence observed at the colony level.

### 2.2. Escovopsioides nivea Strains on Escovopsis Group Phylogeny

The Maximum Likelihood (ML) phylogenetic reconstruction successfully resolved all included genera into independent, well-defined clusters (Figure 1). The major attine-associated lineages (*Escovopsis*, *Manidigitorum*, *Sympodiorosea*, and *Luteomyces*) formed distinct monophyletic clades with maximum or near-maximum bootstrap support (UFBoot = 98–100%). The outgroup taxa (*Cladobotryum*, *Hypomyces*, *Protocrea*, *Neogamszarella*, *Sphaerostilbella*, and *Trichoderma*) effectively rooted the topology, cleanly separating non-attine hypocrealean fungi from the symbiotic genera.

The two novel sequences from Panama nested directly within the *Escovopsioides* cluster alongside four reference sequences, forming a monophyletic clade with maximum bootstrap support (UFBoot = 100%). The topology also recovered *Escovopsioides* as sister to *Luteomyces* with strong support (UFBoot = 89%). Within the *Escovopsioides* clade, the Panamanian samples grouped most closely with *E. nivea* (MF116029), confirming a strong phylogenetic affinity to this species (Figure 1). Additional metadata of strains are provided in Table S1.

### 2.3. Antagonistic Interaction of Escovopsioides and Leucoagaricus

Intraspecific clonal interactions within *L. gongylophorus* strain showed no antagonistic behavior. Moreover, intraspecific clonal interactions among *E. nivea* strain exhibited contact inhibition after 10 days, although physical contact between colonies was established in both groups [27]. In contrast, in interspecific interactions, pairing *L. gongylophorus* with *E. nivea* resulted in an inhibition zone of 0.85 ± 0.17 cm (mean ± standard deviation) between the two cultures. This inhibition zone indicates a clear antagonistic response between *L. gongylophorus* and *E. nivea*, consistent with previously reported antagonistic interactions between these fungal species [27,28,29].

### 2.4. Chemical Characterization of Secondary Metabolites from E. nivea

**Figure 2:**
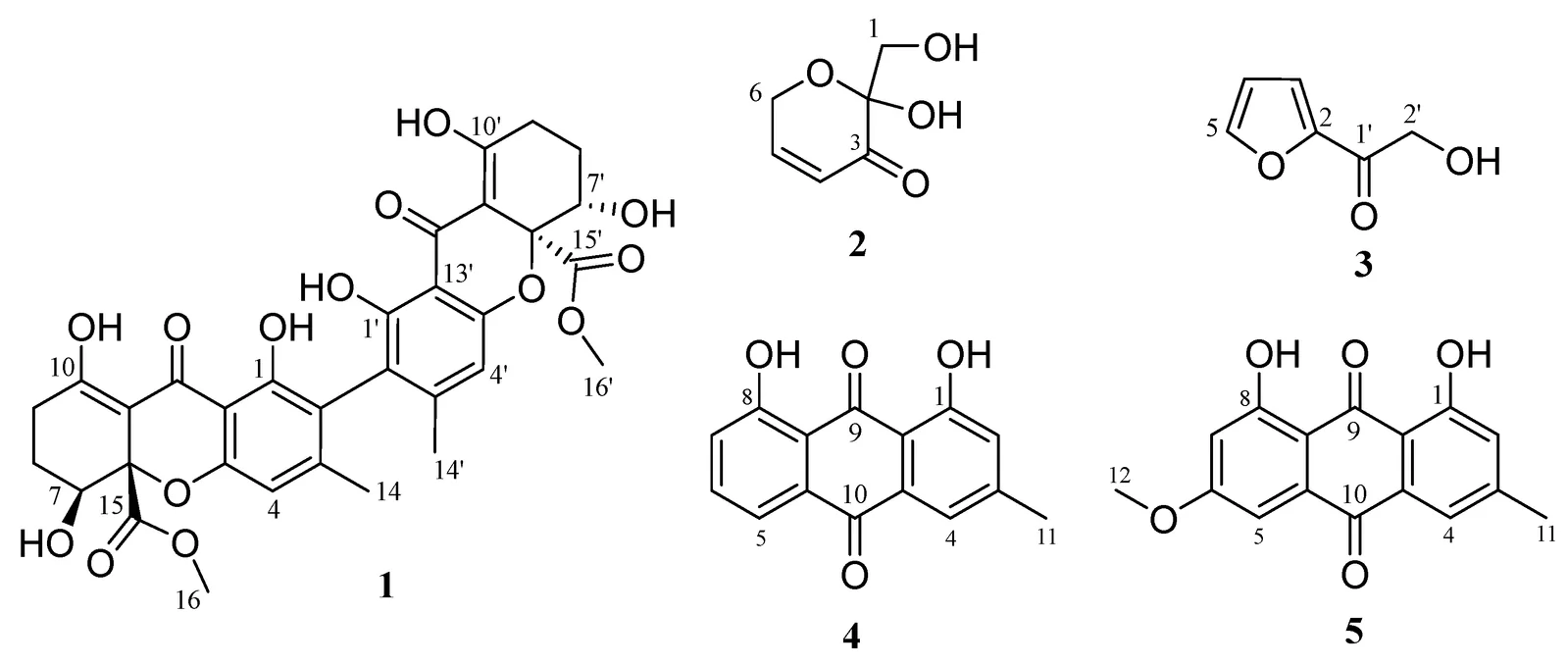
Compounds isolated from *Escovopsioides nivea*.

*E. nivea* was cultured on Potato Dextrose Agar (PDA) to study its major secondary metabolites that could be involved in the antagonistic effects against the fungal cultivar of *A. octospinosus*. We identified five compounds (Figure 2).

Compound **1** was obtained as a yellow solid. The ^13^C NMR spectrum showed sixteen signals [δ_C_ 186.8 (C-12), 177.8 (C-10), 170.4 (C-15), 159.5 (C-1), 158.0 (C-5), 150.4 (C-3), 116.9 (C-2), 109.3 (C-4), 105.0 (C-13), 101.2 (C11), 84.4 (C-6), 72.1 (C-7), 53.4 (C-16), 27.7 (C-9), 23.9 (C-8), and 21.1 (C-14)] (Figure S1). The mass spectrum showed the presence of a protonated molecule [M + H]^+^ = 639 (Figure S2), corresponding to the formula C_32_H_31_O_14_, indicating that it is a dimer. The ^1^H NMR and *g*HSQC spectra showed the presence of two methyl hydrogens [δ_H_ 3.75 (H-16) and 2.05 (H-14)], one aromatic methine hydrogen [δ_H_ 6.55 (H-4)], one oxymethine hydrogen [δ_H_ 4.31 (H-7)], two methylene hydrogen groups [δ_H_ 2.60-2.74 (H-9), and 2.19 and 2.10 (H-8)], and two hydroxyl groups [δ_H_ 13.88 (10-OH) and 11.51 (1-OH)] (Figures S3 and S4). The planar structure was established through the correlations observed in the *g*COSY and *g*HMBC spectra (Figures S5 and S6). Based on these data and comparison with previously published studies (Table S2), compound **1** was identified as Rugulotrosin A [35]. This compound belongs to the subgroup of dimeric tetrahydroxanthones and was discovered from the culture of a *Penicillium* sp. fungus isolated from soil [36]. Biological studies showed that it has significant antibacterial activity against *Bacillus subtilis*, *Enterococcus faecalis*, and *Bacillus cereus* [36].

Compound **2** was obtained as an amorphous colorless solid. The ^13^C NMR spectrum showed six signals [δ_C_ 193.22 (C-3), 150.43 (C-5), 125.58 (C-4), 96.88 (C-2), 64.85 (C-1), and 61.08 (C-6)] (Figure S7). The ^1^H NMR and *g*HSQC spectra showed the presence of two alkene methine hydrogens [δ_H_ 7.20 (H-5) and 6.07 (H-4)] and two methylene hydrogens [δ_H_ 4.37 and 4.67 (H-6), and 3.52 and 3.92 (H-1)] (Figures S8 and S9). The planar structure was established through the correlations observed in the *g*COSY and *g*HMBC spectra (Figures S10 and S11). The mass spectrum showed the presence of a cationized molecule [M + Na]^+^ = 167.0315 corresponding to the formula C_6_H_8_O_4_Na (Figure S12). Based on these data and comparison with previously published studies (Table S3), compound **2** was identified as microthecin [37]. It has been reported from species of the genus *Morchella* [38,39] and from the red alga *Gracilariopsis lemaneiformis* [37] and exhibits antibacterial activity of 100–200 ppm against Gram-positive and Gram-negative bacteria, including *Pseudomonas aeruginosa*, but is inactive against yeasts and mold, and presents cytotoxicity against some mammalian cell lines [40].

Compound **3** was obtained as a brown amorphous solid. The ^13^C NMR spectrum showed six signals corresponding to the following carbons [δ_C_ 189.5 (C-1’), 151.9 (C-2), 119.0 (C-3), 113.4 (C-4), 148.6 (C-5), and 65.8 (C-2’)] (Figure S13). The ^1^H NMR and *g*HSQC spectra showed the presence of a methylene hydrogen [δ_H_ 4.69 (H-2’)], and three aromatic methine hydrogens [δ_H_ 7.79 (H-5), 7.39 (H-3), and 6.65 (H-4)] (Figures S14 and S15). The planar structure was established through the correlations observed in the *g*COSY and *g*HMBC spectra (Figures S16 and S17). The mass spectrum showed the presence of a protonated molecule [M + H]^+^ = 127 corresponding to the formula C_6_H_7_O_3_ and a cationized molecule [M + Na]^+^ = 149 corresponding to the formula C_6_H_6_O_3_Na (Figure S18). Based on these data and comparison with previously published studies (Table S4), compound **3** was identified as 2-(1-oxo-2-hydroxyethyl) furan [41]. This compound has been isolated from fungi such as *Cornynascus setosus* [41], *Rhizoctonia solani* [42], and species of *Morchella* [38,43]. The 2-(1-oxo-2-hydroxyethyl) furan was reported with strong antiradical activity [43].

Compound **4** was obtained as an orange-yellow solid. The ^13^C NMR spectrum displayed fifteen signals [δ_C_ 192.7 (C-9), 182.2 (C-10), 162.9 (C-1), 162.6 (C-8), 149.5 (C-3), 137.1 (C-6), 133.8 (C-5a), 133.4 (C-4a), 124.7 (C-7), 124.5 (C-2), 121.5 (C-4), 120.1 (C-5), 116.0 (C-8a), 113.9 (C-1a), and 22.4 (C-11)] (Figure S19). The ^1^H NMR and *g*HSQC spectra showed eight signals assigned to 5 aromatic methine hydrogens [δ_H_ 7.82 (H-5), 7.67 (H-6), 7.65 (H-4), 7.29 (H-7), and 7.10 (H-2)], one methyl hydrogen [δ_H_ 2.46 (H-11)], and two phenolic hydroxyl group hydrogens [δ_H_ 12.1 (8-OH) and 12.0 (1-OH)] (Figures S20 and S21). The planar structure was established through the correlations observed in the *g*COSY and *g*HMBC spectra (Figures S22 and S23). The mass spectrum showed the presence of a protonated molecule [M + H]^+^ = 255 corresponding to the formula C_15_H_11_O_4_ (Figure S24). Based on these data and comparison with previously published studies (Table S5), compound **4** was identified as chrysophanol [44]. It has been observed in different plant families, including *Polygonaceae*, *Rhammnaceae*, *Fabaceae*, *Liliaceae*, *Asphodelaceae*, *Euphorbiaceae*, *Meliaceae*, *Podocarpaceae*, *Picramniaceae*, and *Hemerocallidaceae* [45]. Compound **4** has a wide spectrum of biological activity, such as anticancer, hepatoprotective, neuroprotective, anti-inflammatory, anti-ulcer, and antibacterial properties [45]. Compound **4** was first reported in fungi from *Penicillium isladicum* Sopp (also called *Talaromyces islandicus*) [46].

Compound **5** was obtained as an orange-yellow solid. The NMR data of **5** (Figures S25–S29) are similar to those of **4**. The main difference observed was a methoxy group on carbon C-6. The mass spectrum showed the presence of a protonated molecule [M + H]^+^ = 285 corresponding to the formula C_16_H_13_O_5_ (Figure S30). Based on these data and comparison with previously published studies (Table S6), compound **5** was identified as physcion [44]. It has been isolated from plants of the following genera: *Rheum*, *Rhamus*, *Polygonum*, *Frangula*, *Muehlenbeckia*, *Reynoutria*, *Anethum*, *Vismia*, *Ventilago*, *Cassia*, *Rumex*, and *Osmunda*, as well as from marine fungus *Microsporum* sp., and sponge-associated fungus *Eurotium cristatum* [47]. Like the structurally similar compound **4**, compound **5** has diverse biological activities [47].

### 2.5. Antagonist Bioassay of Compounds isolated from E. nivea Against L. gongylophorus

**Figure 3:**
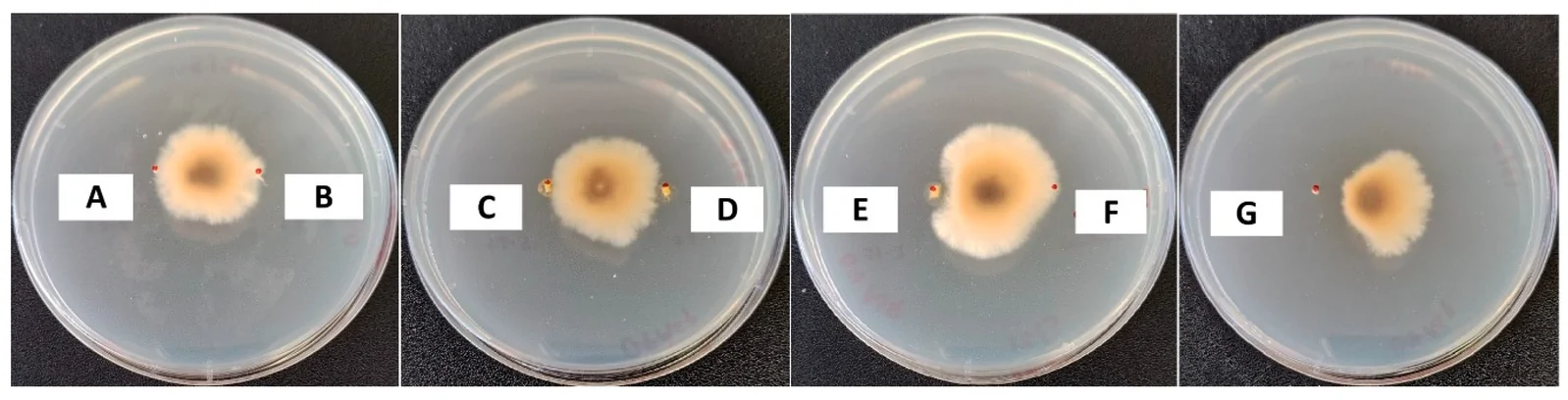
Antagonist bioassay of compounds **1**–**5** (100 µg) against *L. gongylophorus*: (**A**) microthecin (**2**), (**B**) 2-(1-oxo-2-hydroxyethyl) furan (**3**), (**C**) physcion (**5**), (**D**) chrysophanol (**4**), (**E**) rugulotrosin A (**1**), (**F**) DMSO as negative control, (**G**) Nystatin (50 µg) as positive control.

The five compounds isolated and identified from *E. nivea* were tested against *L. gongylophorus* on potato dextrose agar (PDA; Figure 3). We observed no zone of inhibition for the following compounds: microthecin (0 mm, N = 1), 2-(1-oxo-2-hydroxyethyl) furan (0 mm, N = 1), and the negative control, DMSO (0 mm, N = 3). In contrast, the following compounds exhibited inhibitory activity: physcion (1.50 ± 0.55 mm, N = 3), chrysophanol (1.35 ± 0.37 mm, N = 3), rugulotrosin A (2.10 ± 1.12 mm, N = 3), and nystatin (50 µg) as a positive control (3.60 ± 2.91 mm, N = 3) (data are presented as mean ± standard deviation, and number of replicates). Compounds microthecin and 2-(1-oxo-2-hydroxyethyl) furan were inactive, physcion and chrysophanol showed slight activity, and rugulotrosin A exhibited the highest inhibition zone, although it was less than that of the positive control, Nystatin. Compounds physcion and chrysophanol have a similar structural skeleton to emodin, previously isolated from *Escovopsis weberi* [31]. Emodin was reported to have antifungal activity against *L. gongylophorus*, using the agar diffusion assay [31], suggesting that physcion and chrysophanol can also be active. These results support that *E. nivea* can produce a mixture of polyketides, such as rugulotrosin A, physcion and chrysophanol, to control the growth of *L. gongylophorus* in *A. octospinosus* colony.

## 3. Experimental Section

### 3.1. Fungal Isolation and Pure Cultures of E. nivea

Between 2022 and 2025, 20 colonies of *A. octospinosus* (9°07′09.1″ N, 79°42′20.5″ W) and 8 colonies of *M. mikromelanos* (8°19′32.9″ N, 80°11′73.3″ W) were collected in Panama to isolate *E. nivea*, as species within the genus *Escovopsioides* have previously been reported in Brazil [19]. To isolate *E. nivea*, 0.5 g portions of fungal garden material were weighed in humid chambers after removing all ants. These sub-samples were placed individually in Petri dishes (100 × 15 mm) lined with water-moistened paper wipes (Kimwipes^®^) and sealed with Parafilm^®^ [27]. The plates were incubated for 7 days to allow fungal development and initial identification of *E. nivea*. Fungal isolates displaying morphological characteristics consistent with *Escovopsioides* were recovered from the fungal gardens of six *A. octospinosus* nests and two *M. mikromelanos* nests.

Following microscopic identification, small fragments of mycelium were subcultured under sterile conditions in a biosafety cabinet onto Potato Dextrose Agar (PDA) placed at the center of fresh Petri dishes using sterile forceps. The plates were sealed with Parafilm^®^ and incubated at 20 °C for 20 days. Cultures were monitored regularly to ensure pure growth free of contamination. Two isolated strains were sequenced using the internal transcribed spacer (ITS) region to molecularly confirm their identity as *E. nivea*.

### 3.2. Fungal Interaction Assays

To evaluate fungal growth dynamics and competitive interactions, five Petri dishes containing antibiotic-free agar per treatment were used to set up three interaction types: (i) Intraspecific clonal interaction (*L. gongylophorus*): Two plugs of pure *L. gongylophorus* culture placed 6.5 cm apart. (ii) Interspecific interaction (*L. gongylophorus* vs. *E. nivea*): One plug of *L. gongylophorus* and one plug of *E. nivea* placed 6.5 cm apart. And, (iii) Intraspecific clonal interaction (*E. nivea*): Two plugs of pure *E. nivea* culture placed 6.5 cm apart. Ten days later, we recorded the distance in cm between both fungal.

### 3.3. Molecular Identification of E. nivea

#### 3.3.1. DNA Extraction

Genomic DNA was extracted from fungal mycelium using a modified CTAB protocol based on Möller et al. [48]. Briefly, mycelial samples were homogenized with a sterile pestle in 500 µL of CTAB buffer (Alpha Teknova, Inc., Hollister, CA, USA) and incubated at 65 °C for 30 min. Phase separation was performed by adding 400 µL of phenol:chloroform:isoamyl alcohol, mixing thoroughly, and centrifuging at 14,000 rpm for 5 min. The aqueous supernatant (250 µL) was recovered, mixed with an equal volume of isopropanol, and incubated at −20 °C for 30 min to precipitate DNA. After centrifugation (14,000 rpm, 7 min), the pellet was washed with 800 µL of ice-cold 70% ethanol, air-dried, and resuspended in 30 µL of ultrapure water. DNA concentration and purity were measured using a NanoDrop spectrophotometer, and samples were stored at −20 °C until further analysis.

#### 3.3.2. PCR Amplification

The nuclear ribosomal internal transcribed spacer region (ITS1–5.8S–ITS2) was amplified using universal fungal primers ITS1 (5′-TCCGTAGGTGAACCTGCGG-3′) and ITS4 (5′-TCCTCCGCTTATTGATATGC-3′). Amplification was carried out in a total reaction volume of 25 µL containing 12.25 µL of H_2_O, 0.5 µL of each primer (10 µM), 2.5 µL of 10× Reaction Buffer, 3.0 mM MgCl_2_, 0.2 µL (0.5 µL) dNTPs (10 mM), 5.0 µL Q-Solution, 0.25 µL Taq DNA Polymerase, and 0.5 µL of template DNA. Thermal cycling conditions consisted of an initial denaturation at 95 °C for 5 min; followed by 30 cycles of 95 °C for 30 s, 60 °C for 30 s, and 72 °C for 90 s; and a final extension at 72 °C for 10 min. Extraction and PCR negative controls were included in all runs.

Amplicons were separated by electrophoresis on a 2.0% (*w*/*v*) agarose gel in 1× TAE buffer at 94 V for 30 min, alongside a molecular weight marker (GelPilot, QIAGEN, Germantown, MD, USA). Bands were visualized under UV transillumination and photographed.

#### 3.3.3. Purification, Sequencing, Bioinformatic Analysis, and GenBank Accession Numbers

PCR products were purified using the QIAquick PCR Purification Kit (QIAGEN) according to the manufacturer’s protocol and quantified. Sanger sequencing in both directions (forward and reverse) was performed by Macrogen Inc. (Seoul, Republic of Korea). Raw sequences were edited, assembled, and compared against the NCBI nucleotide database using the Basic Local Alignment Search Tool (BLASTn) to confirm species identity based on percentage similarity. The two sequences of *E. nivea* were deposited in GenBank; the accession numbers are PZ972686 (for SUB16474169 20230228-03_Ao) and PZ972687 (for SUB16474169 20210928-02_Ao).

### 3.4. Phylogenetic Tree

To assess the phylogenetic placement of the two obtained ITS sequences, an initial sequence similarity search was conducted using BLAST + 2.16.0, which indicated an affiliation with *Escovopsioides*. For a more robust phylogenetic reconstruction, a dataset comprising 44 ITS sequences were assembled including the two novel sequences from Panama, 34 reference sequences representing the attine-associated genera *Escovopsis*, *Escovopsioides*, *Manidigitorum*, *Sympodiorosea*, and *Luteomyces*, and 8 outgroup sequences from *Cladobotryum*, *Hypomyces*, *Protocrea*, *Gamszarella*, *Sphaerostilbella*, and *Trichoderma*. Sequence accession numbers and additional metadata are provided in Table S1. We selected outgroup taxa following the benchmark framework of Montoya et al. (2021) [18] to ensure proper tree rooting and evaluate clade monophyly within Hypocreaceae, incorporating updated nomenclature (i.e., *Neogamszarella antillana*, previously *Lecanicillium antillanum*). Sequence accession numbers and additional metadata are provided in Table S1.

Sequences were aligned using MAFFT v7.526 [49], and the resulting alignment was trimmed with trimAl v1.5.rev1 [50]. Maximum Likelihood (ML) phylogenetic reconstruction was performed in IQ-TREE multicore v2.4.0 [51]. The best-fit nucleotide substitution model, TN + F + I + G4, was determined using ModelFinder [52]. Branch support was estimated using 1000 ultrafast bootstrap replicates. The final phylogenetic tree was visualized and edited in FigTree v1.4.4. [53].

### 3.5. Materials and Apparatus for Purification and Chemical Characterization of Compounds **1**–**5**

The compounds **1**–**5** were purified using an HPLC (Waters^®^, including a 2487 dual λ Absorbance Detector and a 1515 Pump) and a Waters XTerra^®^ C18 column (10 µm, 250 × 10 mm). The one- and two-dimensional NMR data were obtained on a Jeol 500 MHz spectrometer. Chemical shifts are reported in parts per million (ppm) relative to the solvent residual peak of chloroform-*d*_1_ (^1^H: 7.26 ppm, singlet; ^13^C: 77.16 ppm, triplet) or methanol-*d*_4_ (^1^H: 3.31 ppm, singlet; ^13^C: 49.00 ppm, septet). The mass spectrometry data of **1**, **3**, **4**, and **5** were acquired with a Waters ACQUITY class-H UPLC^TM^ system using a Shim-pack GISS (1.9 μm) C18, reverse phase LC column, 2.1 × 100 mm, a Waters PDA eλ Detector, and a XEVO^®^ TQD spectrometer (Waters Corporation, Milford, MA, USA) supplied with an electrospray ionization (ESI) source. The HRESIMS data of **2** was acquired using a Vanquish UHPLC system including a quaternary pump, an autosampler, and a column oven coupled to a heated electrospray ionization (ESI) source and a Q-Exactive Focus Orbitrap mass spectrometer (all Thermo Fisher Scientific, Waltham, MA, USA).

### 3.6. Purification of Compound **1**

The fungus was inoculated in 700 mL of PDA (29 g of PDA per liter of distilled water) distributed in 28 Petri dishes of 100 × 20 mm. They were in an incubator at 26 °C in the dark for 14 days. The culture was cut into small squares and placed in a 1 L Erlenmeyer flask, and ethyl acetate was added to cover it, leaving it until the next day. Before filtering, the flask was placed in an ultrasonic bath, and then the solvent was filtered and dried under vacuum using a rotary evaporator. This solid–liquid extraction was performed three times to obtain the crude extract (429.5 mg).

A VLC was used with a glass column (10 cm × 4 cm) and 10 g of silica gel (200–400 mesh) to fractionate the crude extract. The gradient used to fractionate it was as follows: 100 mL [100% Hexane, *FA1*: 120.4 mg]; 100 mL [Hexane:Ethyl Acetate (1:1), *FA2*: 40.3 mg]; 100 mL [100% Ethyl Acetate, *FA3*: 158.7 mg]; 100 mL [Ethyl Acetate:Methanol (1:1), *FA4*: 45.2 mg]; 100 mL [100% MeOH, *FA5*: 38.1 mg].

The fraction *FA3* was injected into an HPLC, using a Waters XTerra^®^ C18 column (10 µm, 250 × 10 mm), with a gradient from A (20% MeOH-H_2_O) to B (100% MeOH), at a flow rate of 1.5 mL/min: min 0–10, an isocratic flow of 20% B; min 10–20, gradient from 20% to 100% B; min 20–35, isocratic flow of 100% B; min 35–36, gradient from 100% to 20% B; min 36–40, isocratic flow of 20% B. Three fractions were obtained: *FA3.1* (min 11, 12.3 mg), *FA3.2* (min 14.5, 3.1 mg), *FA3.3* (min 32.5, rugulotrosin A: 7.8 mg).

### 3.7. Purification of Compounds **2**–**5**

The fungus was inoculated in 5 L of PDA distributed in 20 × 1 L Erlenmeyer flasks. They were kept in an incubator at 26 °C in the dark for 14 days. The culture was dried by lyophilization. The material was placed in two 1 L chemical beakers, ethyl acetate was poured to completely cover the contents, they were sonicated for 10 min, and then the solvent was filtered to dry it under vacuum with a rotavapor to obtain the organic extract. This solid–liquid extraction was repeated 12 times, yielding 3.6 g of the crude extract.

The crude was fractionated by solid phase extraction using an SPE-C18 cartridge (45 µm, 10 g/70 Chromabond). For each gram of extract, the following gradient was made: 100 mL (20% MeOH-H_2_O, *FB1*: 269.8 mg); 100 mL (40% MeOH-H_2_O, *FB2*: 30.3 mg); 100 mL (60% MeOH-H_2_O, *FB3*: 41.8 mg); 100 mL (80% MeOH-H_2_O, *FB4*: 53.8 mg); 100 mL (100% MeOH, *FB5*: 46.9 mg); and 100 mL (100% acetone, *FB6*: 307 mg).

Fraction *FB1* (100 mg) was injected into an HPLC (Waters^®^), using a Waters XTerra^®^ C18 column (10 µm, 250 × 10 mm), with a gradient from A (20% MeOH-H_2_O) to B (100% MeOH), at a flow of 1.5 mL/min: 0–10 min, an isocratic flow of 20% B; 10–20 min, gradient 20% to 100% B; 20–25 min, isocratic flow of 100% B; 25–26 min, gradient from 100% to 20% B; 26–30 min, 20% B isocratic flow. Two fractions were obtained: *FB1.1* (8 min, microthecin: 17.8 mg) and *FB1.2* (11 min, 2-(1-oxo-2-hydroxyethyl) furan: 4.1 mg).

Fraction *FB6* was injected into an HPLC, using a Waters XTerra^®^ C18 column (10 µm, 250 × 10 mm), with a gradient from A (20% MeOH-H_2_O) to B (100% MeOH), at a flow rate of 1.5 mL/min: 0–10 min, an isocratic flow of 95% B; 10–20 min, gradient 95% to 100% B; 20–25 min, isocratic flow of 100% B; 25–26 min, gradient from 100% to 95% B; and 26–30 min, 95% B isocratic flow. Two fractions were obtained: *FB6.1* (15 min, chrysophanol: 3.3 mg) and *FB6.2* (18 min, physcion: 2.4 mg).

### 3.8. Antagonist Bioassay of Compounds **1**–**5** Against L. gongylophorus

Each compound-fungal challenge was replicated three times on Potato Dextrose Agar (PDA). The fungus was placed in the center of PDA Petri dishes (90 × 15 mm) and grown alone for 10 days. Two microliters of compounds (100 µg) were placed 0.5 cm near the edge of the fungus. The positive control used was Nystatin (50 µg). Challenges were monitored during 7 days [10]. 

## 4. Conclusions

The fungus *E. nivea* is phylogenetically related to the specialized pathogen *Escovopsis* and acts as a fungal antagonist against the growth of *L. gongylophorus*. Here, we report for the first time the chemical characterization of secondary metabolites produced by *E. nivea* that exhibit antagonistic activity. Furthermore, we demonstrate that polyketides produced by fungi within the *Escovopsis* group, specifically rugulotrosin A (**1**), chrysophanol (**4**), and physcion (**5**), effectively inhibit the growth of *L. gongylophorus*. These compounds are known for their broad spectrum of biological activities. Compounds **4** and **5** are structural analogues of emodin, a metabolite previously isolated from *E. weberi* with potent inhibitory activity against *L. gongylophorus*. The characterization of these inhibitory secondary metabolites addresses key questions regarding the ecological role of *E. nivea* within the obligate attine ant–basidiomycete mutualism, as well as the evolutionary trajectory of mycoparasitism in the *Escovopsis* group toward mutualistic basidiomycetes. Although recent comparative genomic studies of *Escovopsis* and related genera have identified metabolic pathways for secondary metabolite biosynthesis, in situ verification of compound production has been lacking. Our findings provide foundational data that will facilitate future studies on the biosynthetic pathways and biosynthetic gene clusters (BGCs) of *E. nivea* and related mycoparasites.

## Data Availability

Dataset available upon request from the authors.

## Supplementary Materials

The following supporting information can be downloaded at https://www.mdpi.com/article/10.3390/molecules31213847/s1: Table S1: Metadata of strains used in the phylogenetic analyses; Table S2: NMR data of Rugulotrosin A (**1**) in CDCl_3_ 500 (^1^H) y 125 (^13^C) MHz; Figure S1: ^13^C NMR spectrum of **1** in CDCl_3_ at 125 MHz; Figure S2: Mass spectrum of **1**; Figure S3: ^1^H NMR spectrum of **1** in CDCl_3_ at 500 MHz; Figure S4: *g*HSQC spectrum of **1** in CDCl_3_; Figure S5: *g*COSY spectrum of **1** in CDCl_3_; Figure S6: *g*HMBC spectrum of **1** in CDCl_3_; Table S3: NMR data of microthecin (**2**) in CD_3_OD at 500 (^1^H) and 125 (^13^C) MHz compared with literature in D_2_O at 400 (^1^H) and 100 (^13^C) MHz; Figure S7: ^13^C NMR spectrum of **2** in CD_3_OD at 125 MHz; Figure S8: ^1^H NMR spectrum of **2** in CD_3_OD at 500 MHz; Figure S9: *g*HSQC spectrum of **2** in CD_3_OD; Figure S10: *g*COSY spectrum of **2** in CD_3_OD; Figure S11: *g*HMBC spectrum of **2** in CD_3_OD; Figure S12: HRESIMS of **2**; Table S4: NMR data of 2-(1-oxo-2-hydroxyethyl) furan (**3**) in CD_3_OD at 500(^1^H) and 125(^13^C) MHz compared with data of literature in CDCl_3_ at 400(^1^H) and 100(^13^C) MHz; Figure S13: ^13^C NMR spectrum of **3** in CD_3_OD at 125 MHz; Figure S14: ^1^H NMR spectrum of **3** in CD_3_OD at 500 MHz; Figure S15: *g*HSQC spectrum of **3** in CD_3_OD; Figure S16: *g*COSY spectrum of **3** in CD_3_OD; Figure S17: *g*HMBC spectrum of **3** in CD_3_OD; Figure S18: Mass spectrum of **3**; Table S5: NMR data of chrysophanol (**4**) in CDCl_3_ at 500(^1^H) and 125(^13^C) MHz; Figure S19: ^13^C NMR spectrum of **4** in CDCl_3_ at 125 MHz; Figure S20: ^1^H NMR spectrum of **4** in CDCl_3_ at 500 MHz; Figure S21: *g*HSQC spectrum of **4** in CDCl_3_; Figure S22: *g*COSY spectrum of **4** in CDCl_3_; Figure S23: *g*HMBC spectrum of **4** in CDCl_3_; Figure S24: Mass spectrum of **4**; Table S6: NMR data of physcion (**5**) in CDCl_3_ at 500(^1^H) and 125(^13^C) MHz; Figure S25: ^13^C NMR spectrum of **5** in CDCl_3_ at 125 MHz; Figure S26: ^1^H NMR spectrum of **5** in CDCl_3_ at 500 MHz; Figure S27: *g*HSQC spectrum of **5** in CDCl_3_; Figure S28: *g*COSY spectrum of **5** in CDCl_3_; Figure S29: *g*HMBC spectrum of **5** in CDCl_3_; Figure S30: Mass spectrum of **5**.
