# Supplementary material for: Chemical Characterization of Secondary Metabolites of Escovopsioides nivea and Their Antagonistic Activity Against the Leaf-Cutter Ant Fungal Mutualist Leucoagaricus gongylophorus
Source: Molecules. 2026 Oct 25;31(21):3847. doi: 10.3390/molecules31213847 (PMC13765081; doi:10.3390/molecules31213847)
Supplement: Supplementary file 1 [file molecules-31-03847-s001.zip › molecules-4534057-supplementary.pdf]

Supporting Information

Chemical Characterization of Secondary Metabolites of *Escovopsioides nivea* and Their Antagonistic Activity Against the Leaf-Cutter Ant Fungal Mutualist *Leucoagaricus gongylophorus*

Tania Pérez [1], Belén Z. Hernández [1], Eyleen Vega Gibbs [2], Ricardo Santamaría [3], Daniel Torres-Mendoza [1,4], Marc G. Chevrette [5], Hermógenes Fernández-Marín [6,7,*] and Humberto E. Ortega [1,7,8,*]

1 Laboratorio de Bioorgánica Tropical, Facultad de Ciencias Naturales, Exactas y Tecnología, Universidad de Panamá, Panama 0824, Panama; tania.perez-t@up.ac.pa (T.P.); belen.hernandez@up.ac.pa (B.Z.H.); daniel-t.torres-m@up.ac.pa (D.T.-M.)
2 Escuela de Biología, Facultad de Ciencias Naturales, Exactas y Tecnología, Universidad de Panamá, Panama 0824, Panama; eyleen.vega-g@up.ac.pa
3 División de Bioprospección y Descubrimiento de Fármacos, Instituto de Investigaciones Científicas y Servicios de Alta Tecnología (INDICASAT AIP), Panama 0843-01103, Panama; rsantamaria@indicasat.org.pa
4 Vicerrectoría de Investigación y Postgrado, Universidad de Panamá, Panama 0824, Panama
5 Department of Plant Pathology and Wisconsin Institute for Discovery, University of Wisconsin-Madison, Madison, WI 53715, USA; chevrette@wisc.edu
6 División de Biodiversidad, Instituto de Investigaciones Científicas y Servicios de Alta Tecnología (INDICASAT AIP), Panama 0843-01103, Panama
7 Sistema Nacional de Investigación (SNI), Secretaría Nacional de Ciencia, Tecnología e Innovación, Ciudad del Saber, Clayton, Panama 0816, Panama
8 Departamento de Química Orgánica, Facultad de Ciencias Naturales, Exactas y Tecnología, Universidad de Panamá, Panama 0824, Panama
* Correspondence: hfernandez@indicasat.org.pa (H.F.-M.); humberto.ortegad@up.ac.pa (H.E.O.)

Table S1. Metadata of strains used in the phylogenetic analyses.

Fungal species name	Strain ID	City, State, Country	Habitat	Ant colony ID	GenBank ITS accessions	References
Escovopsioides nivea	CBS 135749ET	Viçosa, Minas Gerais, Brazil	Fungus garden of *Acromyrmex subterraneus subterraneus*		JQ815078	[1]
Escovopsioides nivea	LESF 599	Sentinela do Sul, Rio Grande do Sul, Brazil	Fungus garden of *Acromyrmex heyeri*	AOMB100904-03	MF116028	[2]
Escovopsioides nivea	LESF 601	Rio Claro, São Paulo, Brazil	Fungus garden of *Trachymyrmex* sp.	SES080402-04	MF116029	[2]
Escovopsioides nivea	LESF 1009	Manaus, Amazonas, Brazil	Fungus garden of *Apterostigma* sp.	CALD170307-04	MT273483	[3]
Escovopsis aspergilloides	CBS 423.93ET	Trinidad and Tobago, Trinidad	Fungus garden of *Trachymyrmex ruthae*		NR_137160	[1,3,4]
Escovopsis breviramosa	LESF 055	Camacan, Bahia, Brazil	Fungus garden of *Acromyrmex* sp.	AR110515-01	KM817044	[5,6]
Escovopsis clavata	CBS 145326ET	Florianópolis, Santa Catarina, Brazil	Fungus garden of *Apterostigma* sp.	AR150817-07	MH715096	[3,7]
Escovopsis chlamydosporosa	CBS 149748ET	Novo Airão, Amazonas, Brazil	Fungus garden of *Trachymyrmex* sp. sensu lato	EM170120-01	OQ589809	[6]
Escovopsis diminuta	CBS 149747ET	Novo Airão, Amazonas, Brazil	Fungus garden of *Trachymyrmex* sp. sensu lato	CAR170120-04	MT273476	[3]
Escovopsis elongatistipitata	CBS 149750ET	Novo Airão, Amazonas, Brazil	Fungus garden of *Trachymyrmex* sp. sensu lato	WM170120-03	OQ589831	[6]
Escovopsis gracilis	CBS 149743ET	Camacan, Bahia, Brazil	Fungus garden of *Atta cephalotes*	BMSR120703-01	KM817049	[5,6]
Escovopsis lentecrescens	CBS 135750ET	Viçosa, Minas Gerais, Brazil	Fungus garden of *Acromyrmex subterraneus molestans*		JQ815079	[1,3]
Escovopsis maculosa	CBS 149746ET	Novo Airão, Amazonas, Brazil	Fungus garden of *Acromyrmex* sp.	AR170124-01	MT273475	[3]
Escovopsis moelleri	LESF 320	Viçosa, Minas Gerais, Brazil	Fungus garden of *Acromyrmex subterraneus molestans*		JQ815077	[1,3,8]
Escovopsis multiformis	CBS 145327ET	Florianópolis, Santa Catarina, Brazil	Fungus garden of *Apterostigma* sp	AR 150816-06	MH715091	[3,7]
Escovopsis papillata	CBS 149745ET	Novo Airão, Amazonas, Brazil	Fungus garden of *Apterostigma* sp.	AR170123-01	OQ589840	[6]
Escovopsis peniculiformis	CBS 149744ET	Gamboa, Panama	Fungus garden of *Atta colombica*	NMG010319-22	KM817101	[5,6]
Escovopsis phialicopiosa	CBS 149738ET	Uberlândia, Minas Gerais, Brazil	*Trachymyrmex sp. sensu lato*	SES080922-03	KM817088	[5,6]
Escovopsis pseudocylindrica	CBS 149749ET	Novo Airão, Amazonas, Brazil	Fungus garden of *Trachymyrmex* sp. sensu lato	NMG170120-07	OQ589819	[6]

Escovopsis rectangula	CBS 149739ET	Rondonia, Brazil	Fungus garden of *Acromyrmex* sp.	SES081007-01	KM817091	[5,6]
Escovopsis rosisimilis	CBS 149742TET	Uberlândia, Minas Gerais, Brazil	Fungus garden of *Trachymyrmex* sp. sensu lato	CTL080820-02	KM817086	[5,6]
Escovopsis spicaticlavata	CBS 149740ET	Manaus, Amazonas, Brazil	Fungus garden of *Paratrachymyrmex diversus*	SES090109-04	KM817093	[5,7]
Escovopsis weberi	LESF 146	Corumbataí, São Paulo, Brazil	Fungus garden of *Atta sexdens rubropilosa*		MT273429	[3]
Hypomyces asterophorum	TFC 201316	Madagascar	*Eucalyptus forest*		FN859414	[9]
Hypomyces asterophorum	CBS 67677ET	Japan			FN859395	[9]
Hypomyces samuelsii	TFC 2007-23	Peru	on basidioma of an agaricoid basidiomycete on a stem of a palm		FN859451	[9]
Hypomyces semicirculare	CBS 705. 88ET	Cuba	On old polypore		NR_121425	[9]
Lecanicillium antillanum	CBS 350.85ET	Cuba	on basidioma of an agaricoid		NR_111097	[10]
Luteomyces trichodermoides	CBS137343ET	Rio Claro, São Paulo, Brazil	Fungus garden of *Mycocepurus goeldi*		NR_158383	[3,11]
Luteomyces trichodermoides	LESF 310	Florianópolis, Santa Catarina, Brazil	Fungus garden of *Mycetophylax morschi*	AR140226-04	MH715088	[7]
Luteomyces trichodermoides	LESF 834	Rio Claro, São Paulo, Brazil	Fungus garden of *Mycocepurus smithii*	CR141003-02	MT273455	[3]
Manidigitorum attinorum	CBS 153793ET	Belterra, Pará, Brazil	*Apterostigma urichii*	CALD170314-03	PX225019	[12]
Manidigitorum cervicornutus	CBS 153791ET	Rancho Frio, Darién Province, Panamá	*Apterostigma pilosum group*	UGM030106-02	PX225025	[12]
Manidigitorum minutus	CBS 153792ET	Novo Airão, Amazonas, Brazil	*Apterostigma urichii*	CAR170120-04	PX225022	[12]
Manidigitorum ramosus	LESF 1934	Ribeirão Grande, São Paulo, Brazil	*Apterostigma pilosum group*	DSA240510-01	PX225015	[12]
Manidigitorum sessilis	LESF 875	Bocas del Toro Province, Panamá	*Apterostigma cf. dentigerum*	UGM020602-07	PX225017	[12]
Protocrea pallida	TFC 99209ET	New York, Cleaveland			NR_111329	[13]
Sphaerostilbella aureonitens	GJS 74-87				FJ442633	unpublished
Sympodiorosea kreiselii	LESF 053	Florianópolis, Santa Catarina, Brazil	Fungus garden of *Mycetophylax morschi*	AR090306-01	KJ808767	[8]
Sympodiorosea kreiselii	LESF 302	Florianópolis, Santa Catarina, Brazil	Fungus garden of *Mycetophylax morschi*	AR140227-05	MH715085	[7]
Sympodiorosea kreiselii	LESF 309	Florianópolis, Santa Catarina, Brazil	Fungus garden of *Mycetophylax morschi*	AR140226-05	MT273442	[3]
Sympodiorosea sp.	LESF 864	Iguazu, Argentina	*Mycetarotes parallelus*	SES030331-05	MT273464	[3]
Trichoderma harzianum	CBS 22695	England			AY605713	[14]

Table S2. NMR data of Rugulotrosin A (**1**) in $CDCl_3$ 500 (^1H) y 125 (^{13}C) MHz

No.	Type	δ_C	δ_H, mult. (J, Hz)	δ_C*	δ_H, mult. (J, Hz)*
1/1'	C	159.5		159.4	
1/1'-OH			11.51, s		11.49, s
2/2'	C	116.9		116.8	
3/3'	C	150.4		150.2	
4/4'	CH	109.3	6.55, s	109.2	6.56, s
5/5'	C	158.0		157.9	
6/6'	C	84.4		84.3	
7/7'	CH	72.1	4.31, dd (12.4, 5.1)	72.0	4.32, dd (12.3, 5.0)
8/8'	CH_2	23.9	2.19, m	23.8	2.20, m
			2.10, m		2.10, m
9/9'	CH_2	27.7	2.60-2.74, m	27.5	2.66, m
10/10'	C	177.8		177.6	
10/10'-OH			13.88, s		13.88, s
11/11'	C	101.2		101.1	
12/12'	C	186.8		186.6	
13/13'	C	105.0		104.9	
14/14'	CH_3	21.1	2.05, s	20.9	2.06, s
15/15'	C	170.4		170.2	
16/16'	CH_3	53.4	3.75, s	53.1	3.75, s

*Literature: [15]

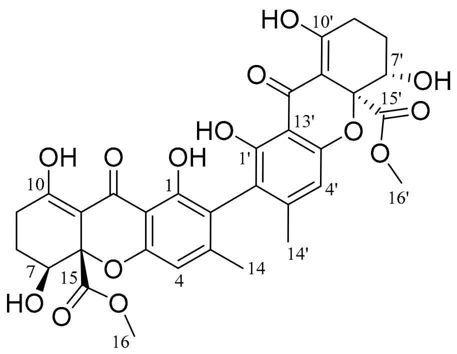

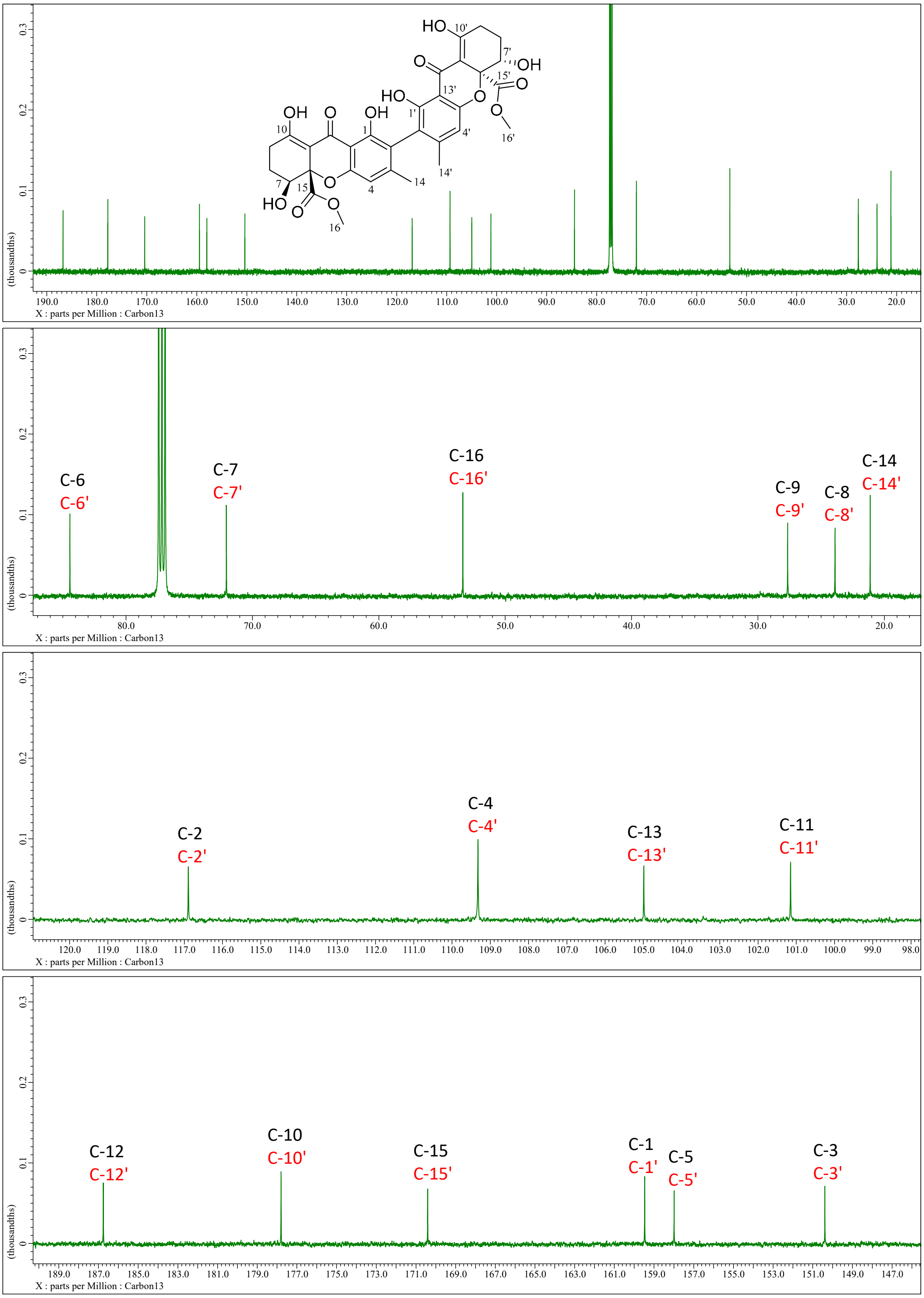

Figure S1. ^{13}C NMR spectrum of **1** in $CDCl_3$ at 125 MHz

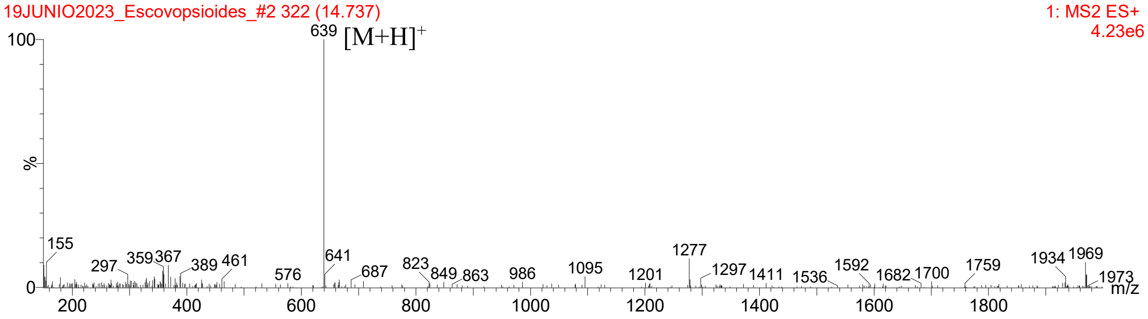

Figure S2. Mass spectrum of **1**

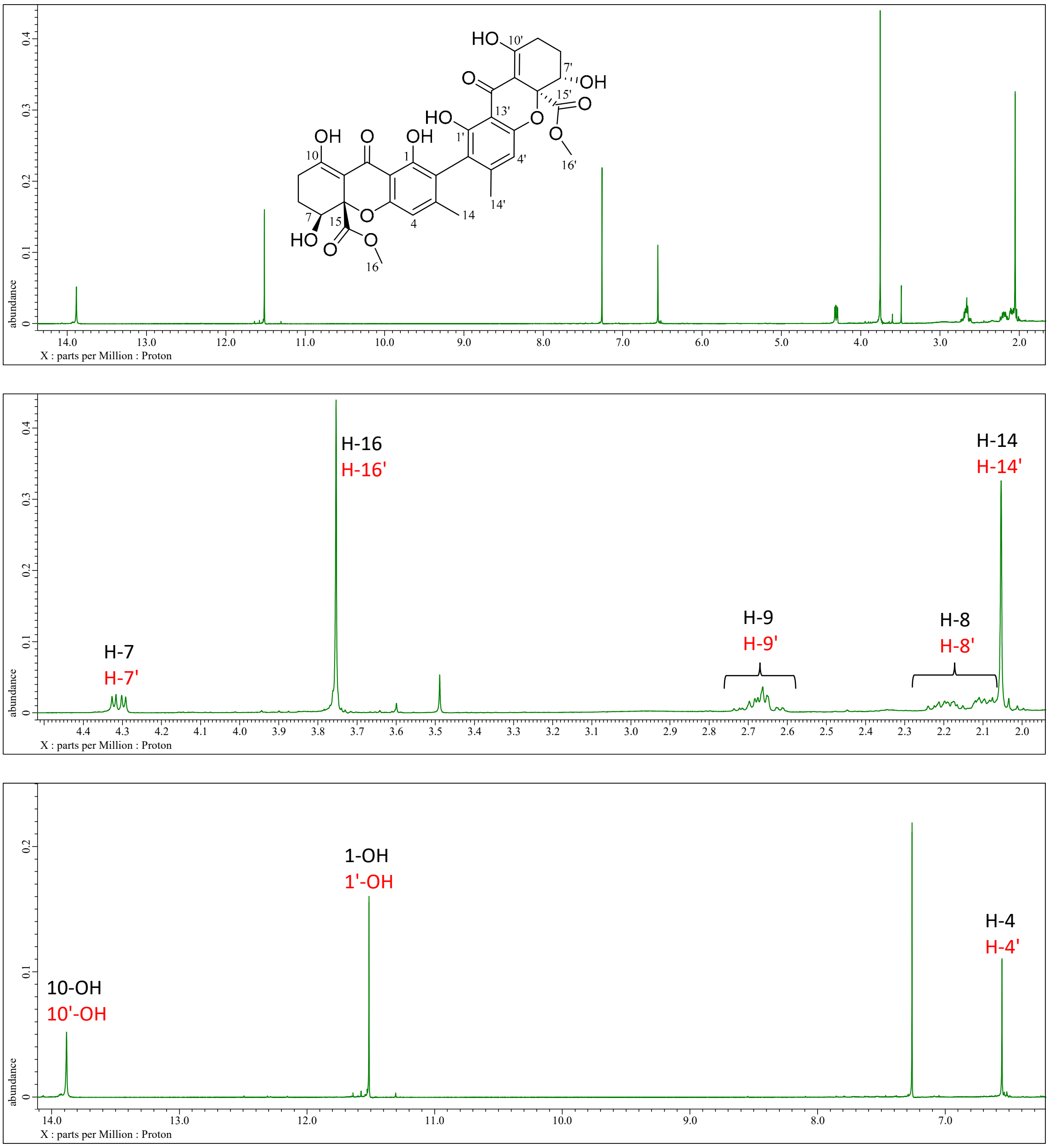

Figure S3. ^{1}H NMR spectrum of **1** in $CDCl_3$ at 500 MHz

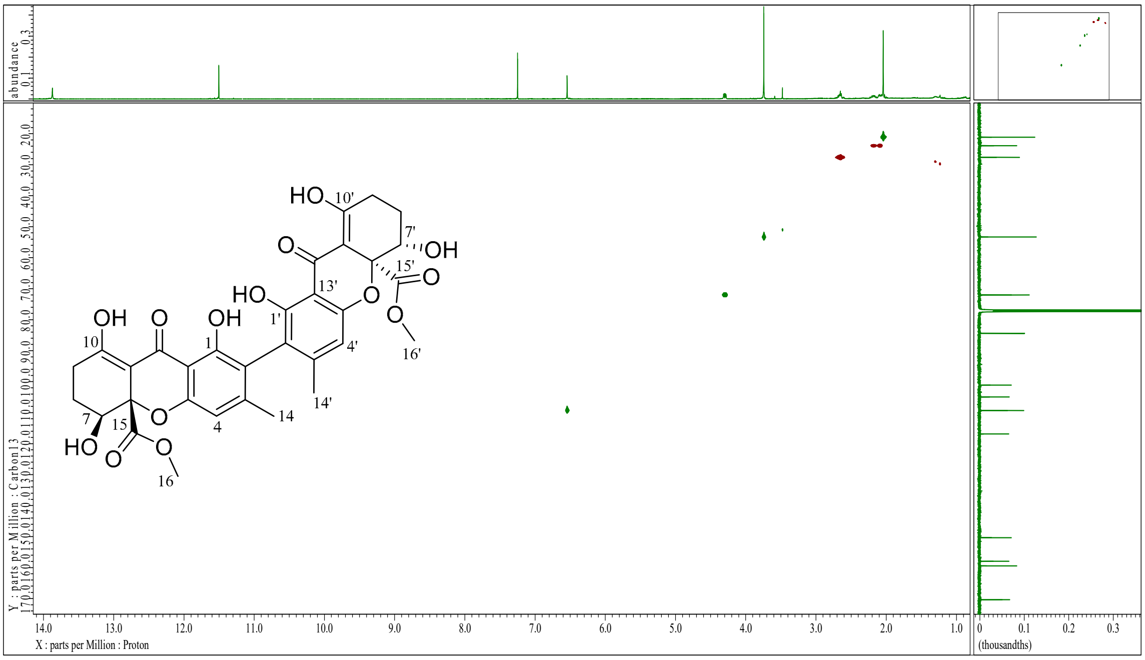

Figure S4. *g*HSQC spectrum of **1** in $CDCl_3$

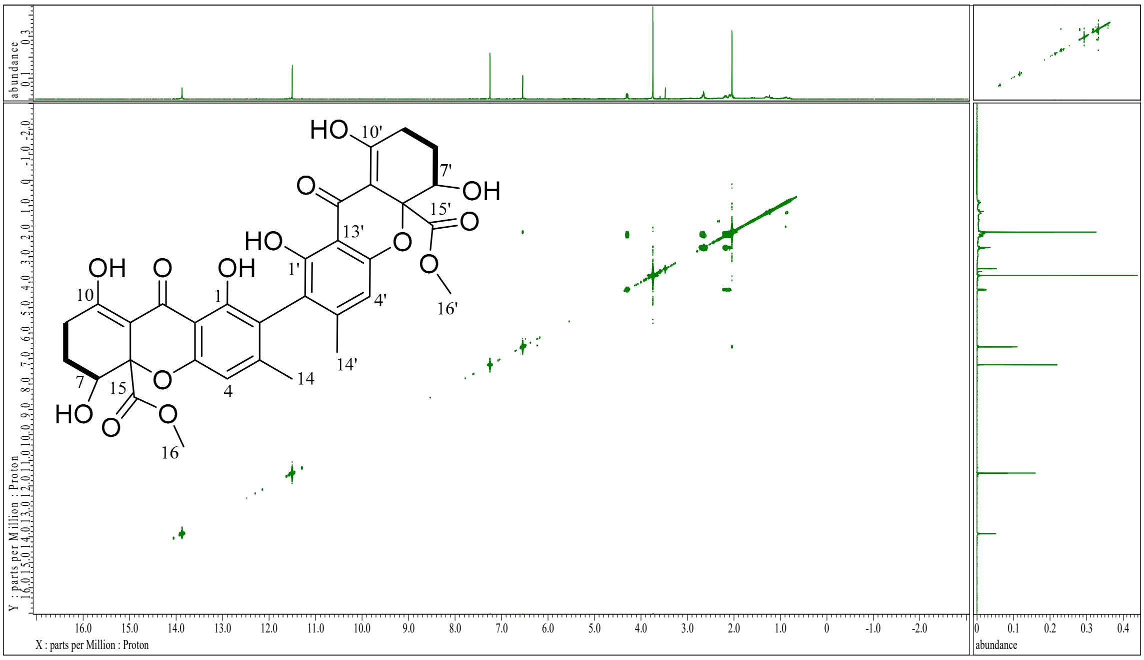

Figure S5. *g*COSY spectrum of **1** in $CDCl_3$

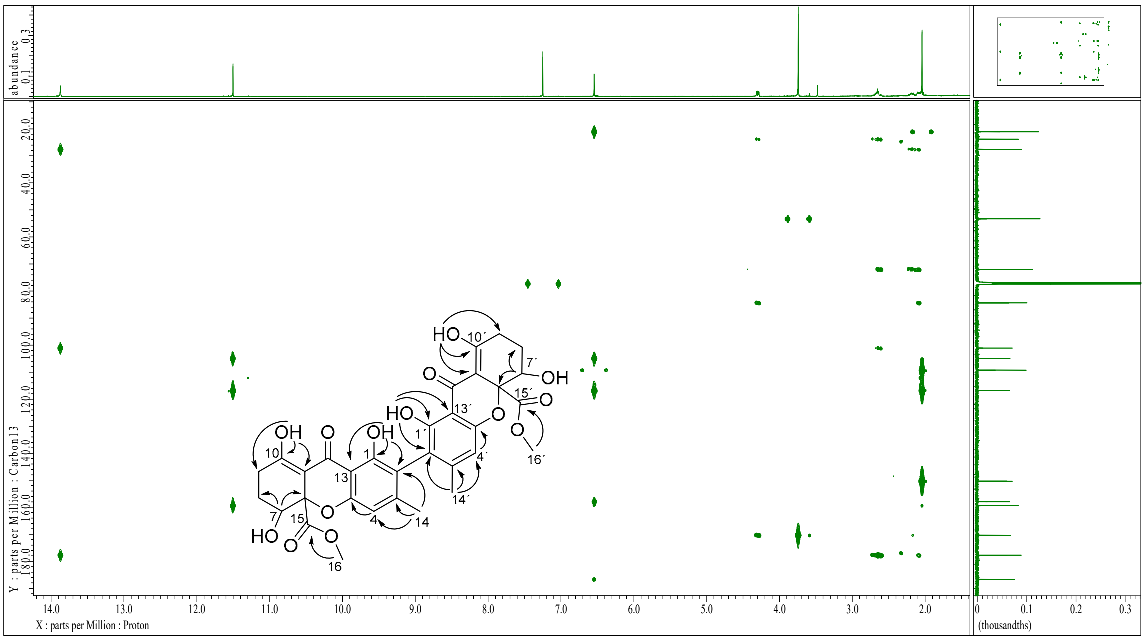

Figure S6. *g*HMBC spectrum of **1** in $CDCl_3$

Table S3. NMR data of microthecin (**2**) in CD_3OD at 500 (^1H) and 125 (^{13}C) MHz compared with literature in D_2O at 400 (^1H) and 100 (^{13}C) MHz

No.	Tipo	δ_C	δ_H, mult. (*J*, Hz)	δ_C*	δ_H, mult. (*J*, Hz)*
1	CH_2	64.85	3.92, d (11.4)	63.20	3.97, d (11.6)
			3.52, d (11.4)		3.66, d (11.6)
2	C	96.88		96.03	
3	C	193.22		193.68	
4	CH	125.58	6.07, ddd (10.4, 2.3, 1.7)	124.21	6.22, ddd (10.4, 2.0, 2.0)
5	CH	150.43	7.20, ddd (10.4, 4.0, 1.7)	152.11	7.38, ddd (4.0, 2.0)
6	CH_2	61.08	4.67, ddd (19.3, 2.3, 1.7)	60.77	4.73. ddd (19.8)
			4.37, ddd (19.3, 4.0, 1.7)		4.53, ddd (19.8)

*Literature: [16]

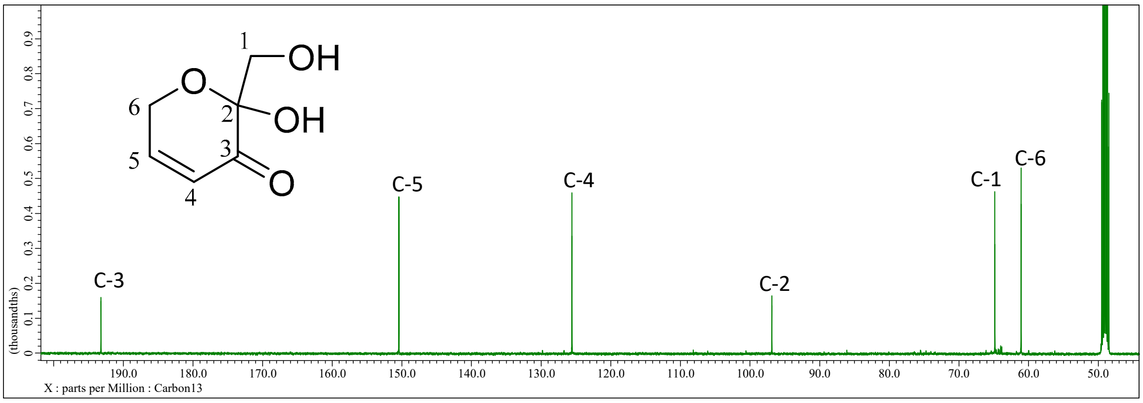

Figure S7. ^{13}C NMR spectrum of **2** in CD_3OD at 125 MHz

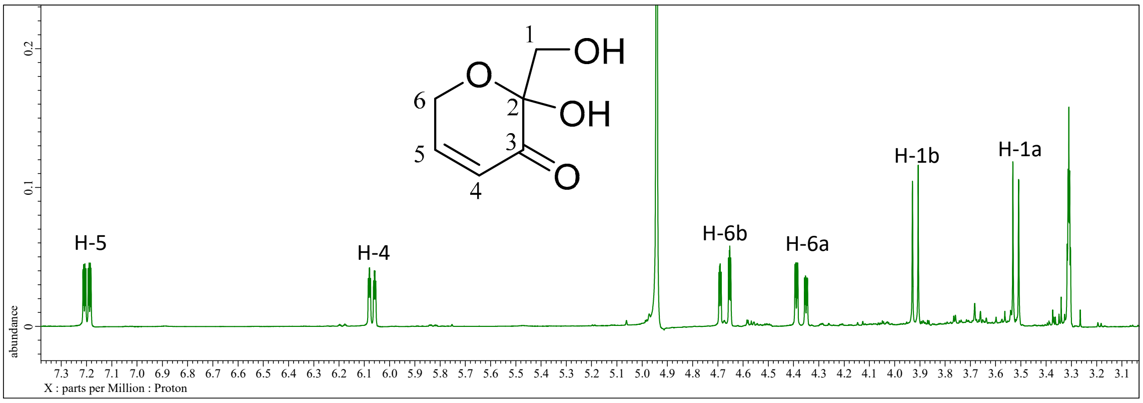

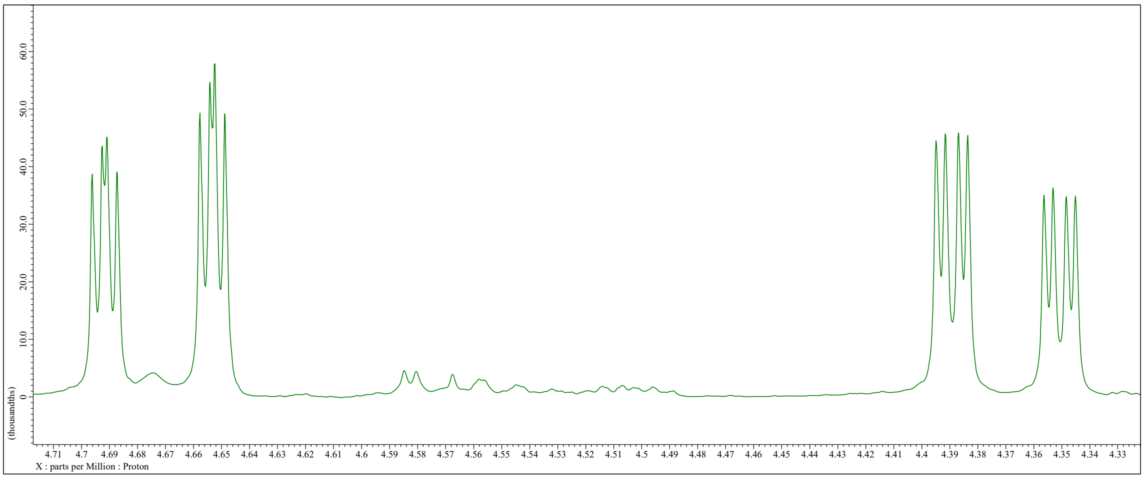

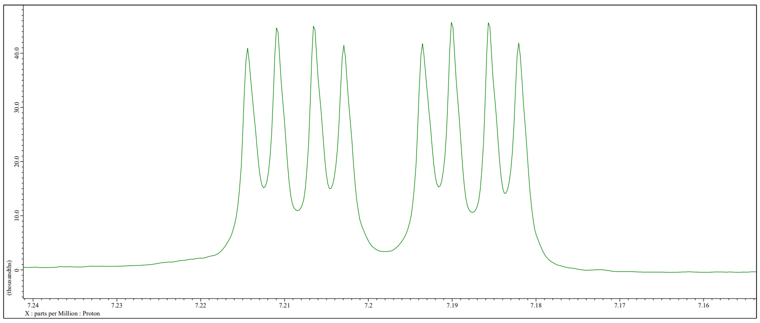

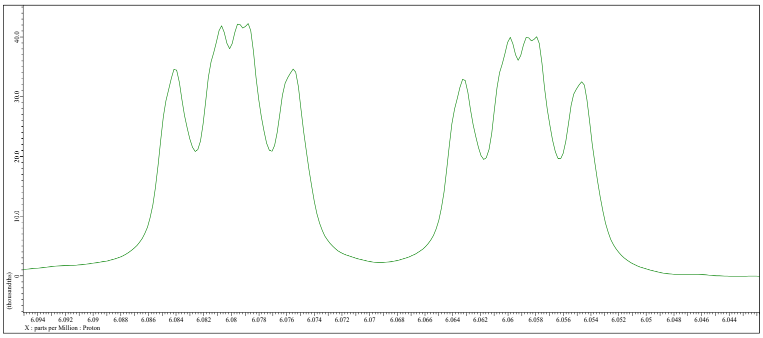

Figure S8. ^{1}H NMR spectrum of **2** in CD_3OD at 500 MHz

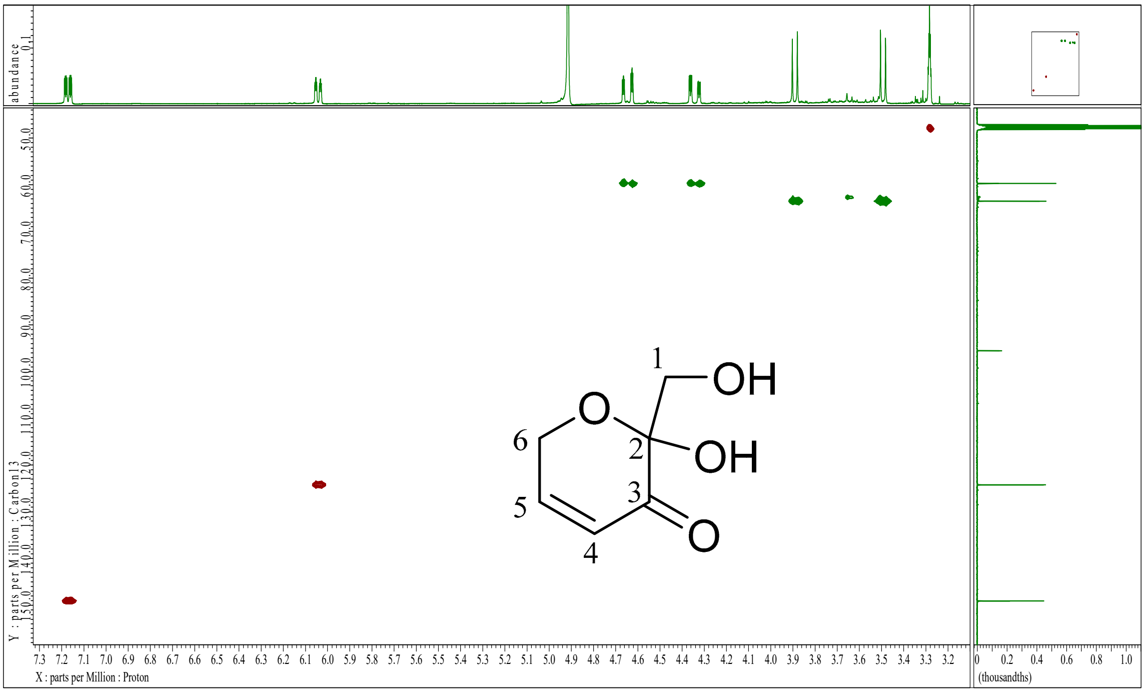

Figure S9. *g*HSQC spectrum of **2** in CD_3OD

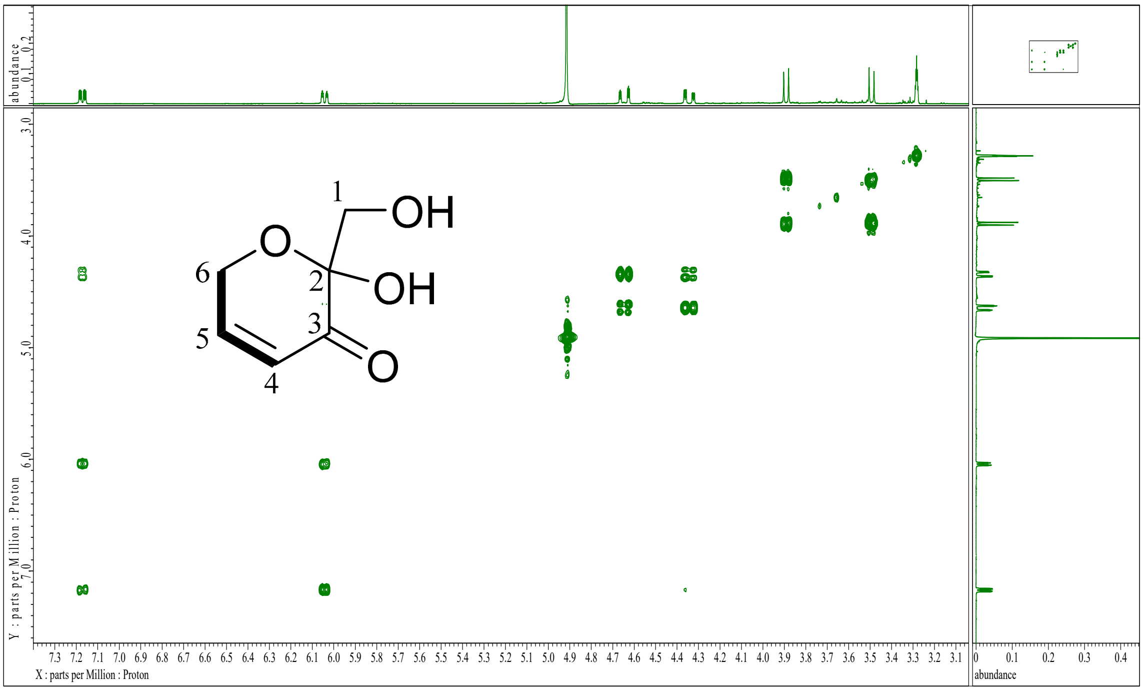

Figure S10. *g*COSY spectrum of **2** in CD_3OD

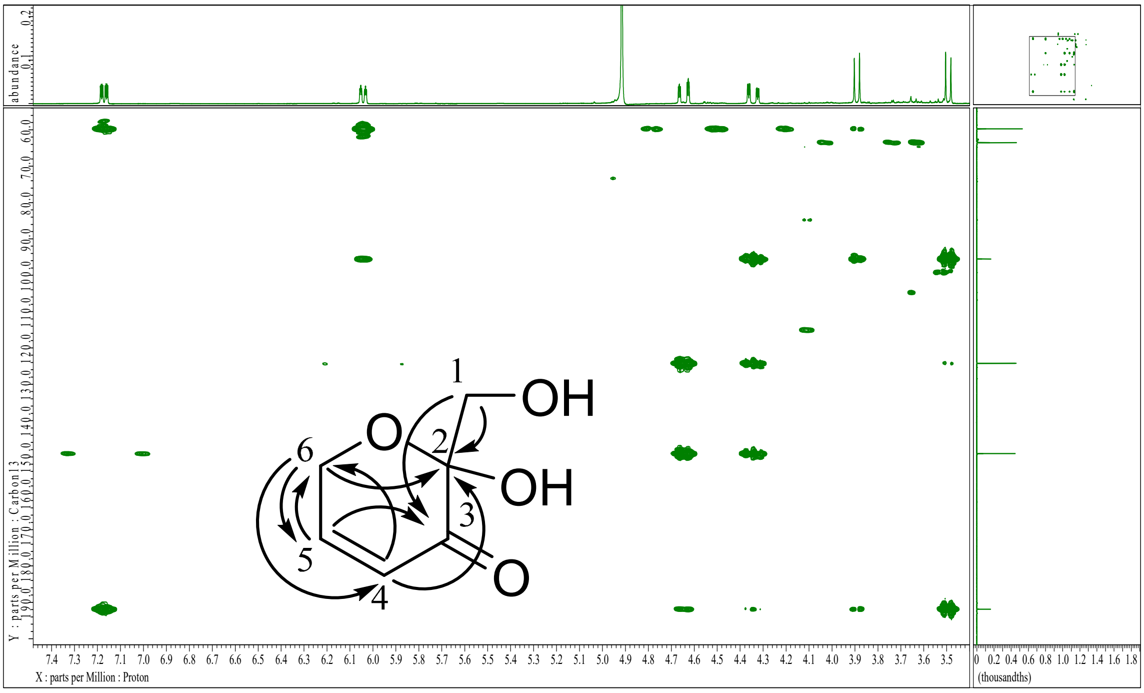

Figure S11. *g*HMBC spectrum of **2** in CD3OD

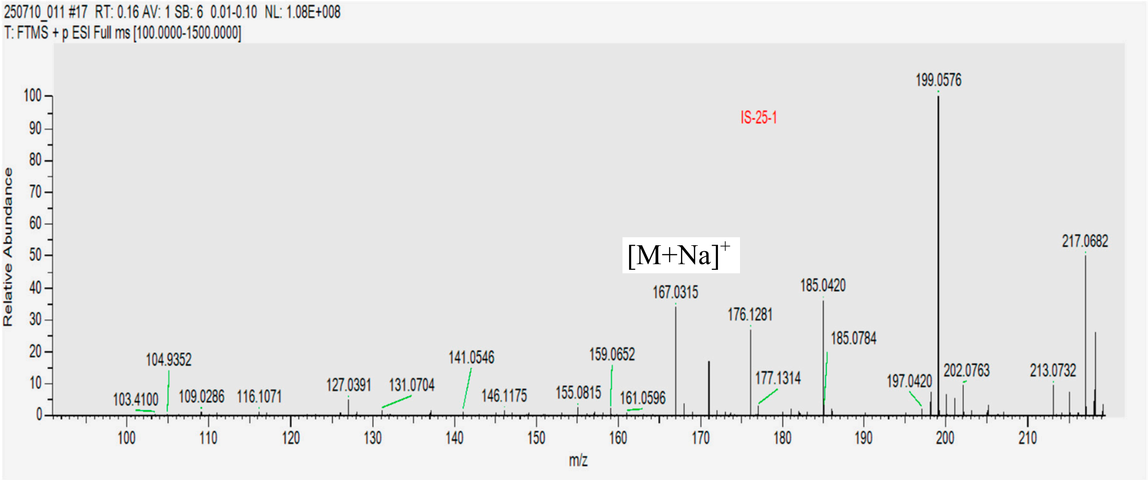

Display Formula	S Fit	RDB	Delta [ppm]	Theo. mass	Combined Score	# Matched Iso.
$C_6H_8O_4{}^{23}Na$	18,35	2,5	0,07	167,03148	84,24	2
$C_4H_3O_2N_6$	12,52	6,5	1,75	167,0312	83,94	1
$C_4H_{11}ON_2{}^{32}S_2$	4,272	0,5	4,55	167,03073	83,5	1

Figure S12. HRESIMS of **2**

Table S4. NMR data of 2-(1-oxo-2-hydroxyethyl) furan (**3**) in CD_3OD at 500(^{1}H) and 125(^{13}C) MHz compared with data of literature in $CDCl_3$ at 400(^{1}H) and 100(^{13}C) MHz

No.	Type	δ_C	δ_H, mult. (*J*, Hz)	δ_C*	δ_H, mult. (*J*, Hz)*
2	C	151.9		150.0	
3	CH	119.0	7.39, dd (3.6, 0.6)	117.9	7.31, dd (3.6, 0.7)
4	CH	113.4	6.65, dd (3.6, 1.7)	112.6	6.61, dd (3.6, 1.7)
5	CH	148.6	7.79, dd (1.7, 0.6)	147.1	7.64, dd (1.7, 0.7)
1'	C	189.5		187.7	
2'	CH_2	65.8	4.69, s	65.1	4.75, d (4.9)

*Literature: [17]

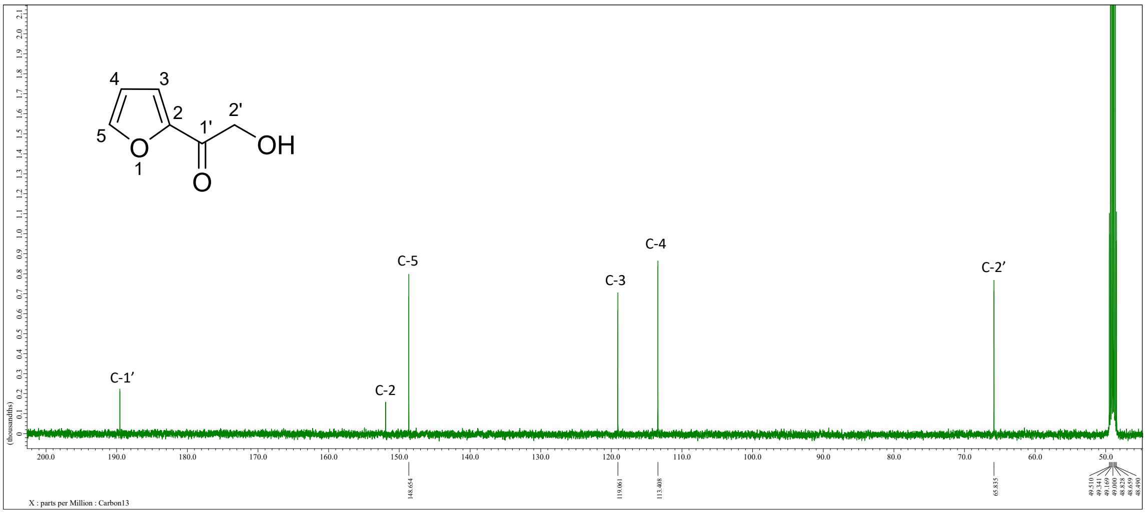

Figure S13. ^{13}C NMR spectrum of **3** in CD_3OD at 125 MHz

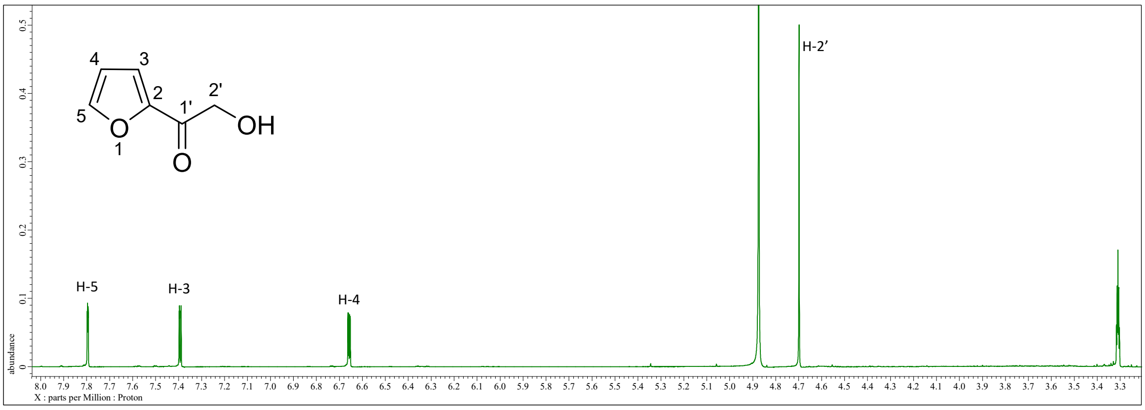

Figure S14. ^{1}H NMR spectrum of **3** in CD_3OD at 500 MHz

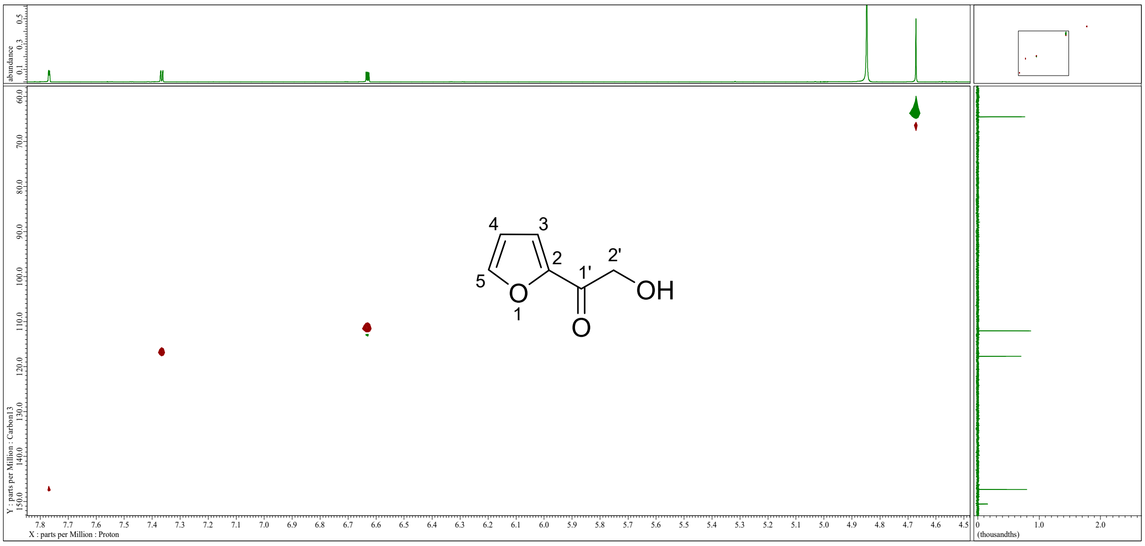

Figure S15. *g*HSQC spectrum of **3** in CD_3OD

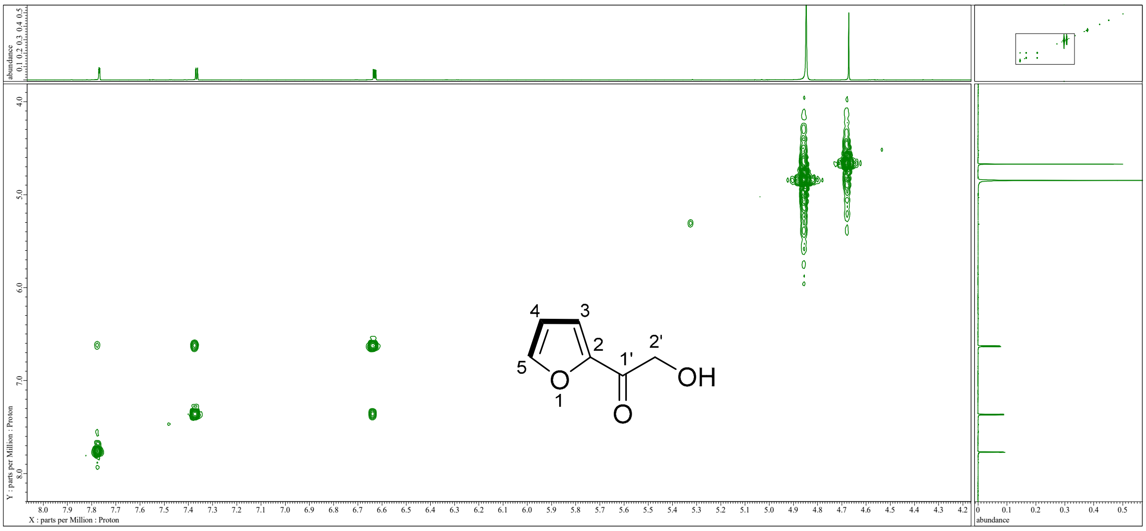

Figure S16. *g*COSY spectrum of **3** in CD_3OD

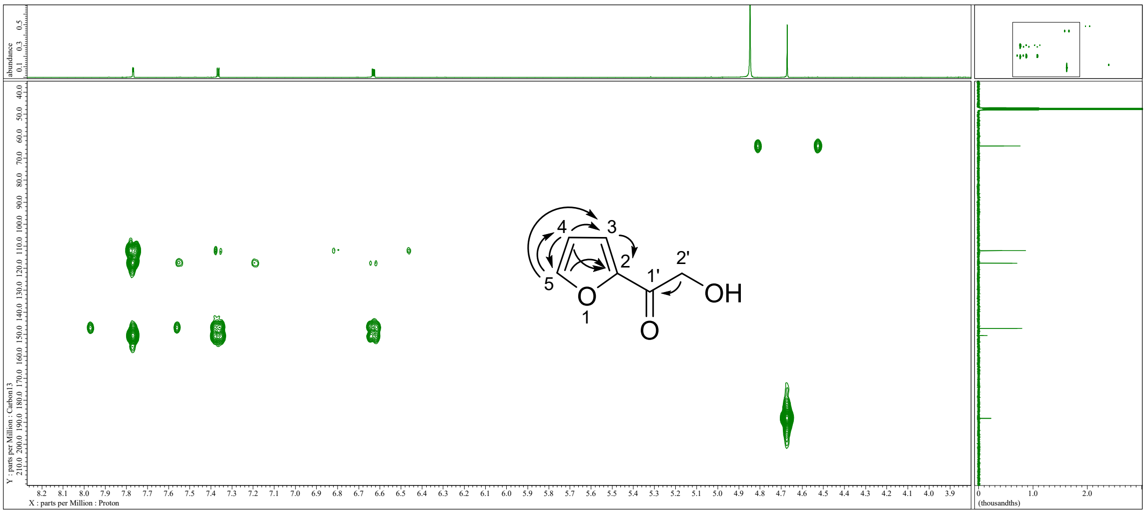

Figure S17. *g*HMBC spectrum of **3** in CD_3OD

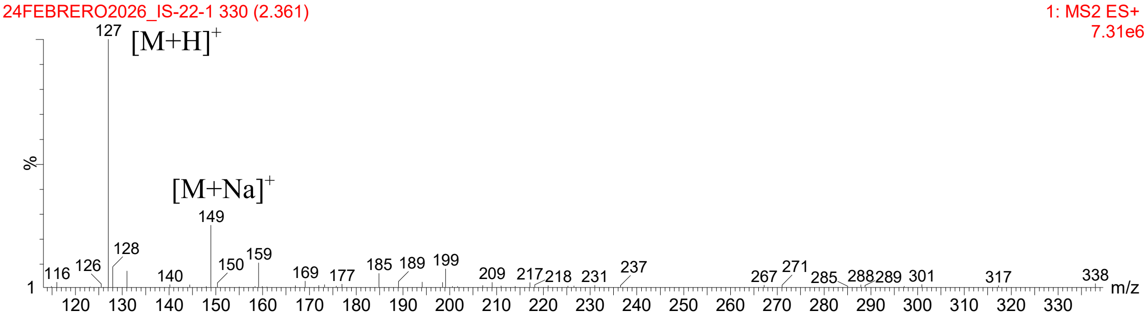

Figure S18. Mass spectrum of **3**

Table S5. NMR data of chrysophanol (**4**) in $CDCl_3$ at 500(^{1}H) and 125(^{13}C) MHz

No.	Type	δ_C	δ_H, mult. (*J*, Hz)	δ_C*	δ_H, mult. (*J*, Hz)*
1	C	162.9		161.9	
1a	C	113.9		113.3	
2	CH	124.5	7.10, brs	124.0	7.08, brs
3	C	149.5		149.0	
4	CH	121.5	7.65, brs	120.9	7.70, brs
4a	C	133.4		132.5	
5	CH	120.1	7.82, dd (7.5, 1.2)	119.5	7.80, dd (7.4, 1.5)
5a	C	133.8		133.2	
6	CH	137.1	7.67, dd (8.4, 7.5)	136.6	7.67, dd (8.4, 7.4)
7	CH	124.7	7.29, dd (8.4, 1.2)	124.2	7.28, dd (8.4, 1.5)
8	C	162.6		162.2	
8a	C	116.0		115.5	
9	C	192.7		192.0	
10	C	182.2		181.5	
11	CH_3	22.4	2.46, s	22.0	2.47, s
1-OH			12.0, s		11.9, s
8-OH			12.1, s		12.0, s

*Literature: [18]

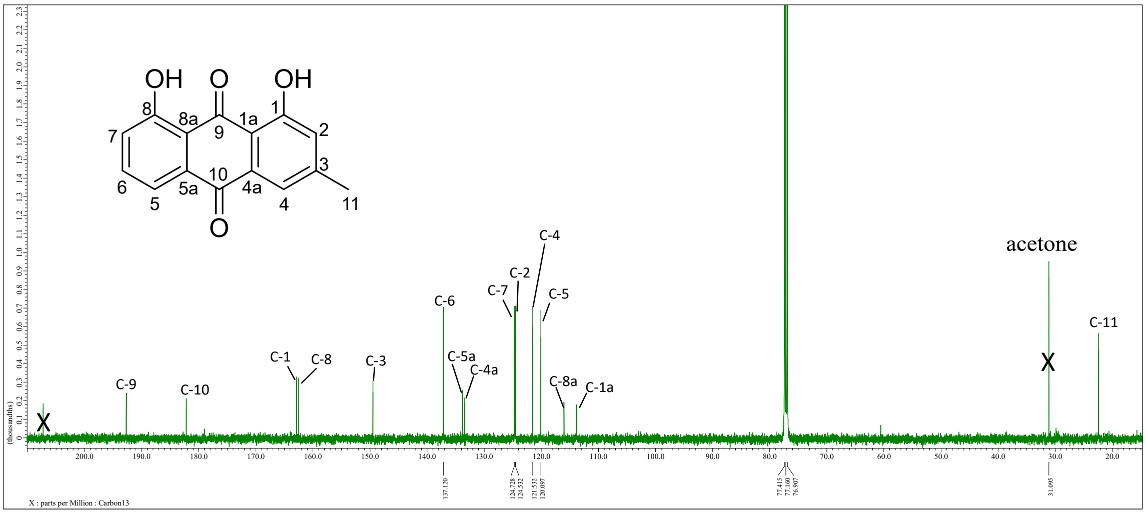

Figure S19. ^{13}C NMR spectrum of **4** in $CDCl_3$ at 125 MHz

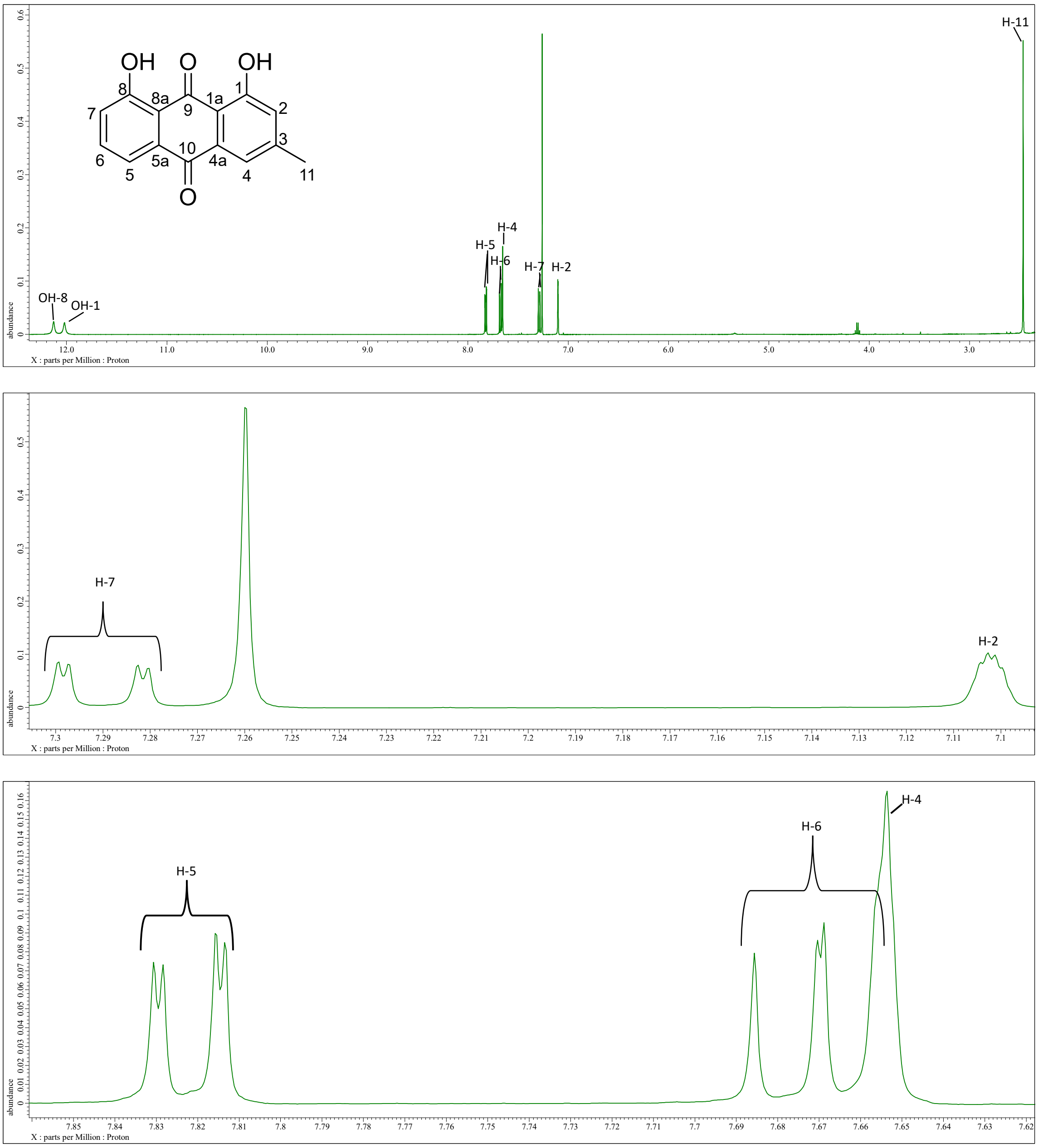

Figure S20. ^{1}H NMR spectrum of **4** in $CDCl_3$ at 500 MHz

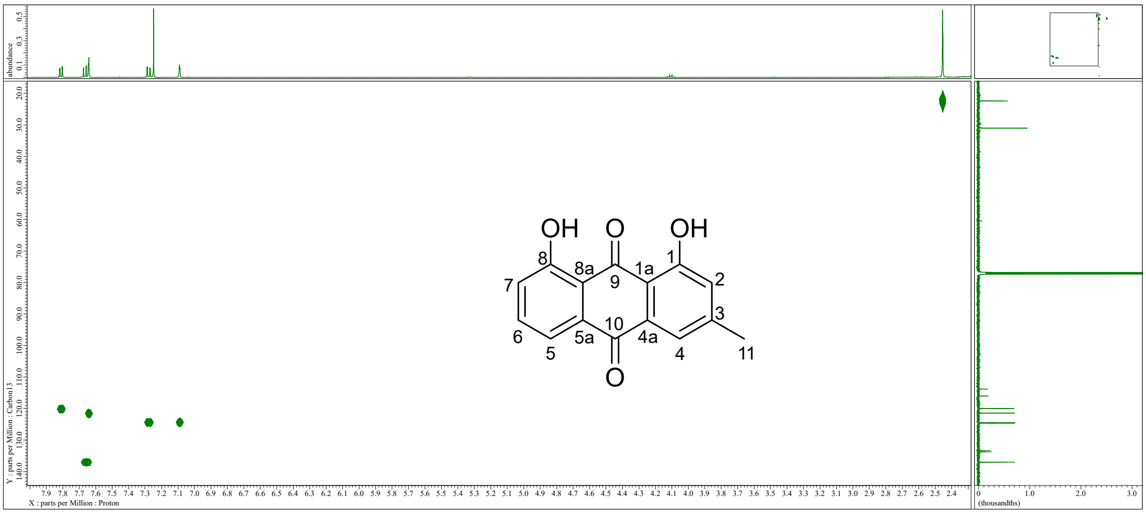

Figure S21. *g*HSQC spectrum of **4** in $CDCl_3$

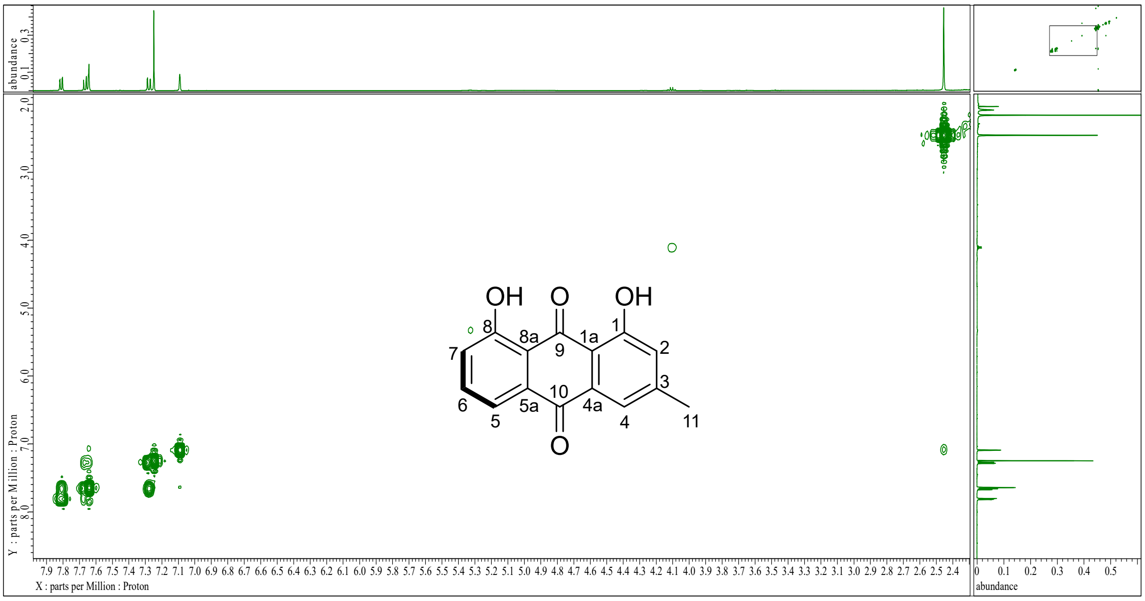

Figure S22. *g*COSY spectrum of **4** in $CDCl_3$

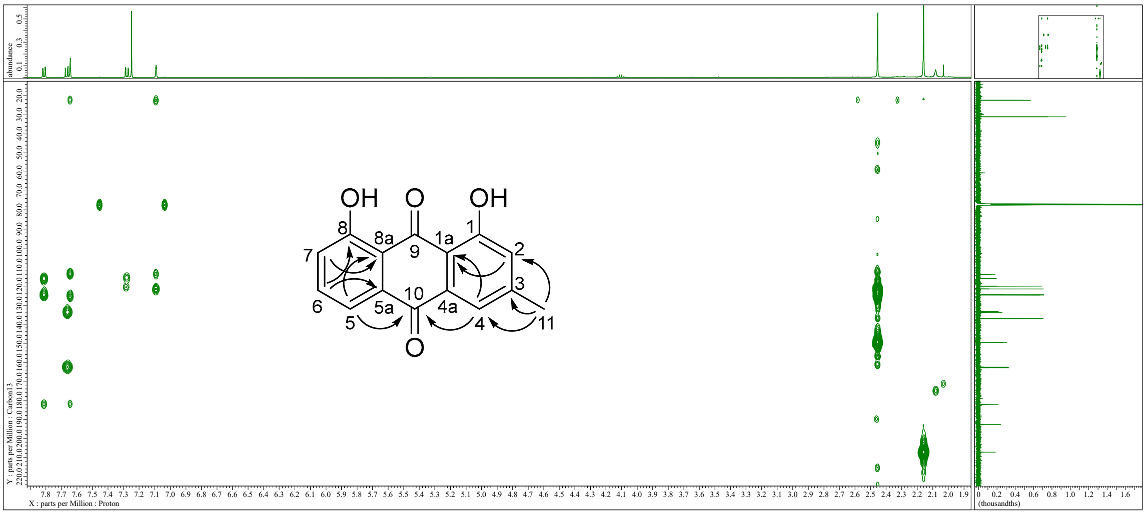

Figure S23. *g*HMBC spectrum of **4** in $CDCl_3$

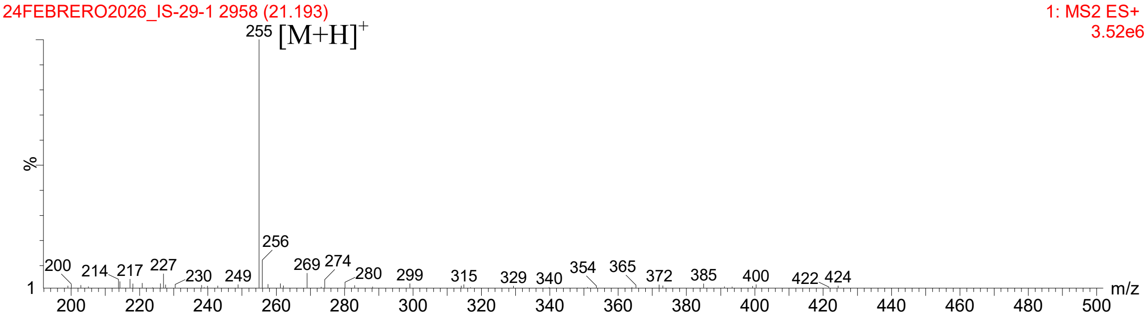

Figure S24. Mass spectrum of **4**

Table S6. NMR data of physcion (**5**) in $CDCl_3$ at 500(^1H) and 125(^{13}C) MHz

No.	Type	δ_C	δ_H, mult. (*J*, Hz)	δ_C*	δ_H, mult. (*J*, Hz)*
1	C	162.6		162.5	
1a	C	113.8		113.7	
2	CH	124.6	7.08, d (1.5)	124.5	7.10, brs
3	C	148.6		148.4	
4	CH	121.4	7.62, d (1.2)	121.3	7.65, brs
4a	C	133.3		133.2	
5	CH	108.3	7.36, d (2.4)	108.2	7.40, d (2.5)
5a	C	135.3		135.2	
6	C	166.6		166.5	
7	CH	106.9	6.68, d (2.4)	106.8	6.71, d (2.5)
8	C	165.3		165.2	
8a	C	110.3		110.3	
9	C	190.8		190.8	
10	C	182.2		182.0	
11	CH_3	22.3	2.44, s	22.1	2.40, s
12	CH_3	56.2	3.93, s	56.0	3.90, s
1-OH			12.12, s		12.15, s
8-OH			12.32, s		12.34, s

*Literature: [18]

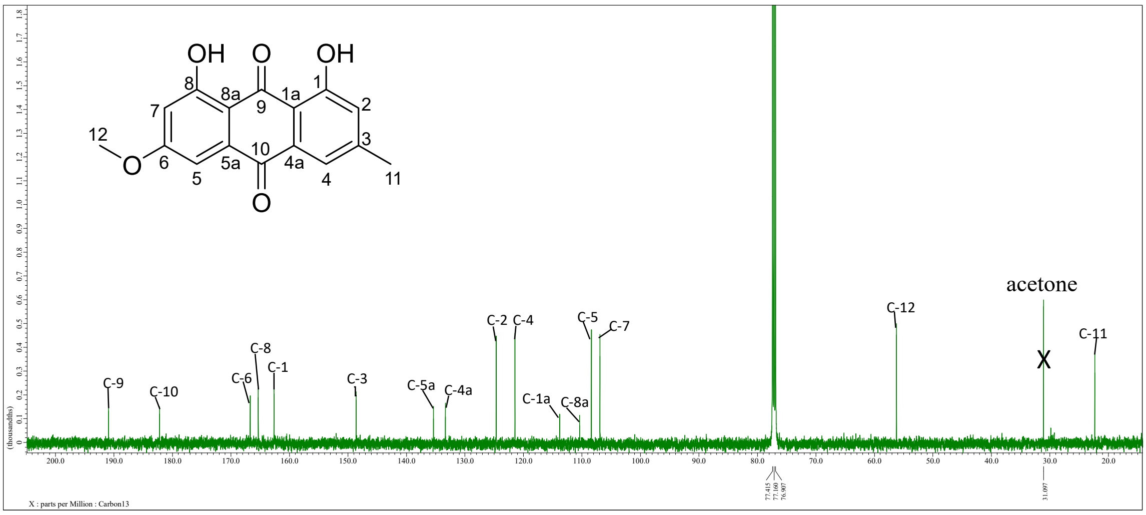

Figure S25. ^{13}C NMR spectrum of **5** in $CDCl_3$ at 125 MHz

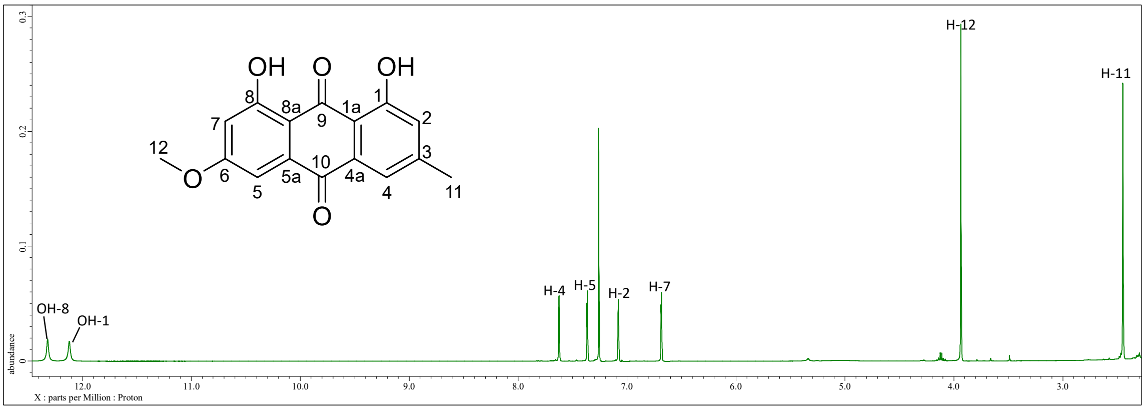

Figure S26. ^{1}H NMR spectrum of **5** in $CDCl_3$ at 500 MHz

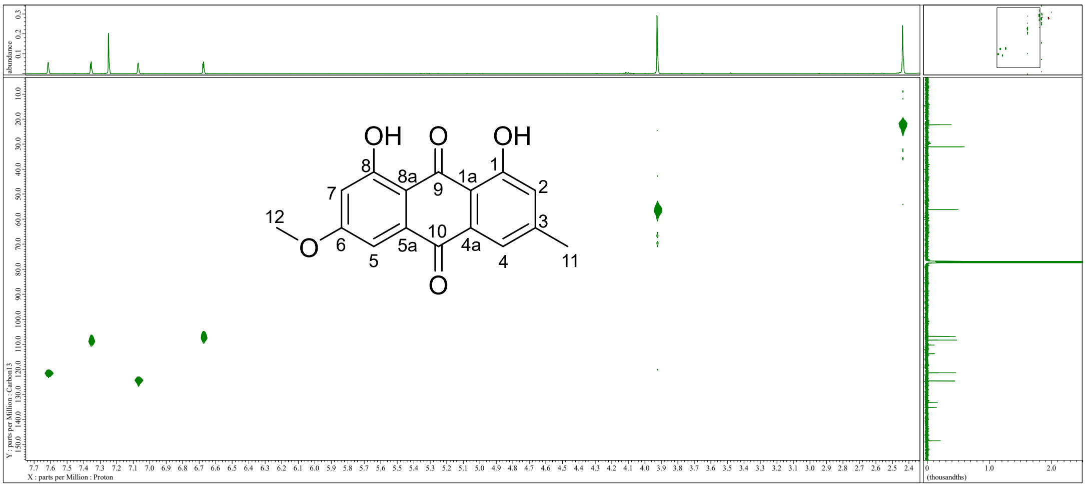

Figure S27. *g*HSQC spectrum of **5** in $CDCl_3$

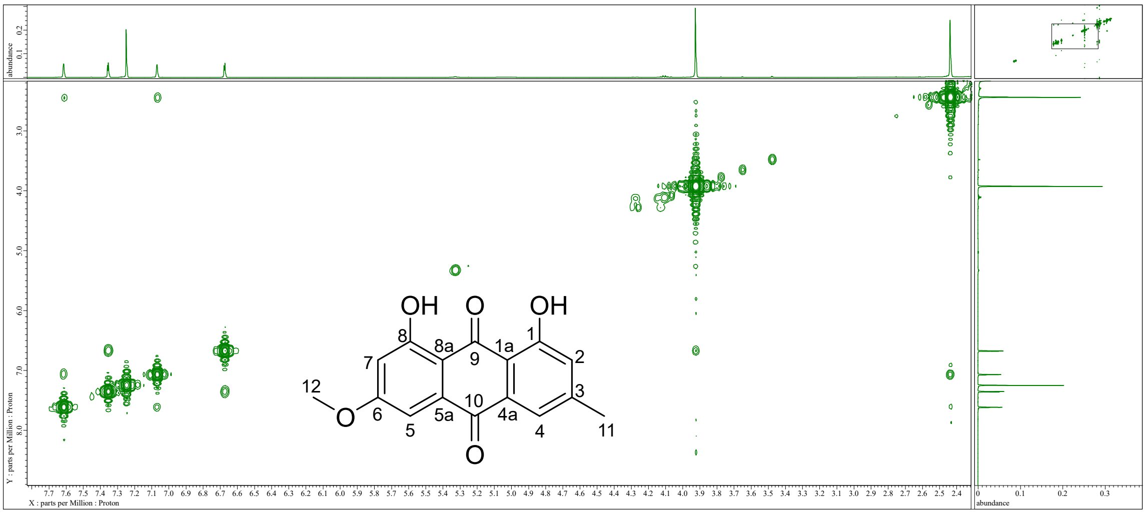

Figure S28. *g*COSY spectrum of **5** in $CDCl_3$

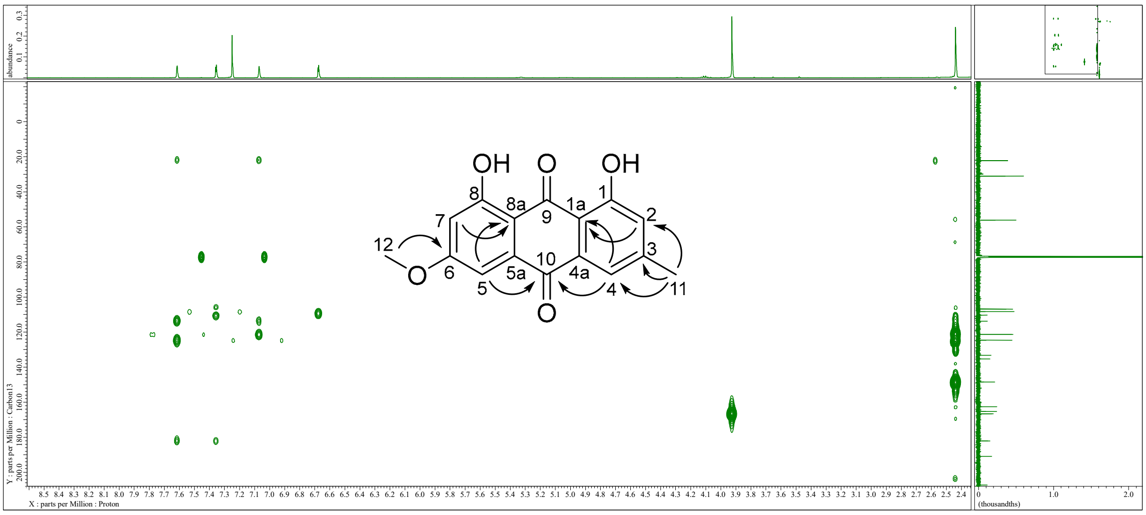

Figure S29. *g*HMBC spectrum of **5** in $CDCl_3$

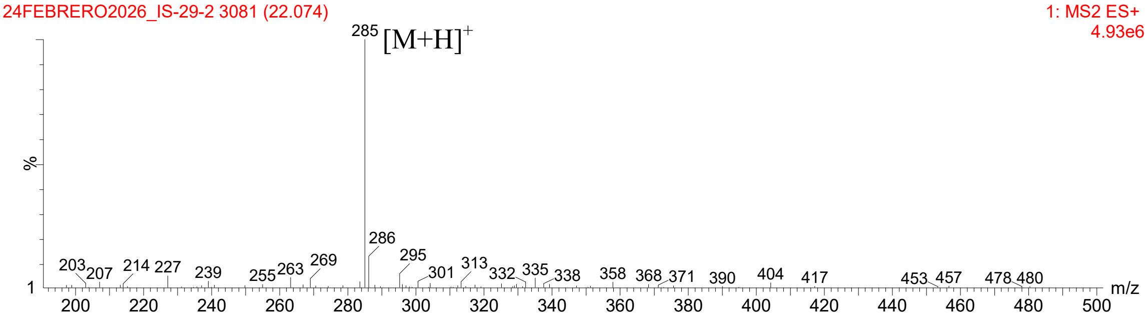

Figure S30. Mass spectrum of **5**

References

1. Augustin, J.O.; Groenewald, J.Z.; Nascimento, R.J.; Mizubuti, E.S.G.; Barreto, R.W.; Elliot, S.L.; Evans, H.C. Yet More “Weeds” in the Garden: Fungal Novelties from Nests of Leaf-Cutting Ants. *PLoS One* **2013**, *8*, 1–17. https://doi.org/10.1371/journal.pone.0082265

2. Osti, J.F.; Rodrigues, A. *Escovopsioides* as a Fungal Antagonist of the Fungus Cultivated by Leafcutter Ants. *BMC Microbiol.* **2018**, *18*, 1–11. https://doi.org/10.1186/s12866-018-1265-x

3. Montoya, Q.V.; Martiarena, M.J.S.; Bizarria, R.; Gerardo, N.M.; Rodrigues, A. Fungi Inhabiting Attine Ant Colonies: Reassessment of the Genus *Escovopsis* and Description of *Luteomyces* and *Sympodiorosea* gens. nov. *IMA Fungus* **2021**, *12*, 1–18. https://doi.org/10.1186/s43008-021-00078-8

4. Currie, C.R.; Wong, B.; Stuart, A.E.; Schultz, T.R.; Rehner, S.A.; Muller, U.G.; Sung, G.-H.; Spatafora, J.W.; Straus, N.A. Ancient Tripartite Coevolution in the Attini Ant-Microbe Symbiois. *Science.* **2003**, *299*, 396–388. DOI: 10.1126/science.1078155

5. Meirelles, L.A.; Solomon, S.E.; Bacci, M.; Wright, A.M.; Mueller, U.G.; Rodrigues, A. Shared *Escovopsis* Parasites between Leaf-Cutting and Non-Leaf-Cutting Ants in the Higher Attine Fungus-Growing Ant Symbiosis. *R. Soc. Open Sci.* **2015**, *2*, 1–11. https://doi.org/10.1098/rsos.150257

6. Montoya, Q.V.; Martiarena, M.J.S.; Rodrigues, A. Taxonomy and Systematics of the Fungus-Growing Ant Associate *Escovopsis* (*Hypocreaceae*). *Stud. Mycol.* **2023**, *106*, 349–397. https://doi.org/10.3114/sim.2023.106.06

7. Montoya, Q.V.; Martiarena, M.J.S.; Polezel, D.A.; Kakazu, S.; Rodrigues, A. More Pieces to a Huge Puzzle: Two New *Escovopsis* Species from Fungus Gardens of Attine Ants. *MycoKeys.* **2019**, *46*, 97–118. https://doi.org/10.3897/mycokeys.46.30951

8. Meirelles, L.A.; Montoya, Q. V.; Solomon, S.E.; Rodrigues, A. New Light on the Systematics of Fungi Associated with Attine Ant Gardens and the Description of *Escovopsis Kreiselii* sp. Nov. *PLoS One*. **2015**, *10*, 1–14. https://doi.org/10.1371/journal.pone.0112067

9. Põldmaa, K. Tropical Species of *Cladobotryum* and *Hypomyces* Producing Red Pigments. *Stud. Mycol.* **2011**, *68*, 1–34. https://doi.org/10.3114/sim.2011.68.01

10. Spatafora, J.W.; Sung, G.H.; Sung, J.M.; Hywel-Jones, N.L.; White, J.F. Phylogenetic Evidence for an Animal Pathogen Origin of Ergot and the Grass Endophytes. *Mol. Ecol.* **2007**, *16*, 1701–1711. https://doi.org/10.1111/j.1365-294X.2007.03225.x

11. Masiulionis, V.E.; Cabello, M.N.; Seifert, K.A.; Rodrigues, A.; Pagnocca, F.C. *Escovopsis trichodermoides* sp. nov., Isolated from a Nest of the Lower Attine Ant *Mycocepurus goeldii*. *Antonie van Leeuwenhoek*. **2015**, *107*, 731–740. https://doi.org/10.1007/s10482-014-0367-1

12. Cruz, M.O. da; Montoya, Q.V.; Gerardo, N.M.; Rodrigues, A. More Diverse than Previously Thought: A Novel *Hypocreaceae* Symbiont from *Apterostigma* Fungus-Farming Ants. *IMA Fungus*. **2026**, *17*, 1–28. https://doi.org/10.3897/imafungus.17.182573

13. Jaklitsch, W.M. European Species of *Hypocrea* Part II: Species with Hyaline Ascospores. *Fungal Diversity.* **2011**, *48*, 1–250. https://doi.org/10.1007/s13225-011-0088-y

14. Chaverri, P.; Castlebury, L.A.; Samuels, G.J.; Geiser, D.M. Multilocus Phylogenetic Structure within the *Trichoderma harzianum*/*Hypocrea lixii* Complex. *Mol. Phylogenet. Evol.* **2003**, *27*, 302–313. https://doi.org/10.1016/S1055-7903(02)00400-1

15. Qin, T.; Skraba-Joiner, S.L.; Khalil, Z.G.; Johnson, R.P.; Capon, R.J.; Porco, J.A. Atropselective Syntheses of (-) and (+) Rugulotrosin A Utilizing Point-to-Axial Chirality Transfer. *Nat. Chem.* **2015**, *7*, 234–240. https://doi.org/10.1038/nchem.2173

16. Broberg, A.; Kenne, L.; Pedersén, M. Presence of Microthecin in the Red Alga *Gracilariopsis lemaneiformis* and Its Formation from 1,5-Anhydro-D-Fructose. *Phytochemistry* **1996**, *41*, 151–154. 10.1016/0031-9422(95)00587-0

17. Fujimoto, H.; Negishi, E.; Yamaguchi, K.; Nishi, N.; Yamazaki, M. Isolation of New Tremorgenic Metabolites from an Ascomycete, *Corynascus setosus*. *Chem. Pharm. Bull. (Tokyo).* **1996**, *44*, 1843–1848. https://doi.org/10.1248/cpb.44.1843

18. Harding, W.W.; Henry, G.E.; Lewis, P.A.; Jacobs, H.; McLean, S.; Reynolds, W.F. Alvaradoins A-D. Anthracenone C Arabinosides from *Alvaradoa jamaicensis*. *J. Nat. Prod.* **1999**, *62*, 98–101. https://doi.org/10.1021/np980300i
